# L_2_-norm multiple kernel learning and its application to biomedical data fusion

**DOI:** 10.1186/1471-2105-11-309

**Published:** 2010-06-08

**Authors:** Shi Yu, Tillmann Falck, Anneleen Daemen, Leon-Charles Tranchevent, Johan AK Suykens, Bart De Moor, Yves Moreau

**Affiliations:** 1 Bioinformatics Group, Department of Electrical Engineering, Katholieke Universiteit Leuven, Kasteelpark Arenberg 10, Heverlee B-3001, Belgium; 2 Systems, Models and Control Group, Department of Electrical Engineering, Katholieke Universiteit Leuven, Kasteelpark Arenberg 10, Heverlee B-3001, Belgium

## Abstract

**Background:**

This paper introduces the notion of optimizing different norms in the dual problem of support vector machines with multiple kernels. The selection of norms yields different extensions of multiple kernel learning (MKL) such as *L*_∞_, *L*_1_, and *L*_2 _MKL. In particular, *L*_2 _MKL is a novel method that leads to non-sparse optimal kernel coefficients, which is different from the sparse kernel coefficients optimized by the existing *L*_∞ _MKL method. In real biomedical applications, *L*_2 _MKL may have more advantages over sparse integration method for thoroughly combining complementary information in heterogeneous data sources.

**Results:**

We provide a theoretical analysis of the relationship between the *L*_2 _optimization of kernels in the dual problem with the *L*_2 _coefficient regularization in the primal problem. Understanding the dual *L*_2 _problem grants a unified view on MKL and enables us to extend the *L*_2 _method to a wide range of machine learning problems. We implement *L*_2 _MKL for ranking and classification problems and compare its performance with the sparse *L*_∞ _and the averaging *L*_1 _MKL methods. The experiments are carried out on six real biomedical data sets and two large scale UCI data sets. *L*_2 _MKL yields better performance on most of the benchmark data sets. In particular, we propose a novel *L*_2 _MKL least squares support vector machine (LSSVM) algorithm, which is shown to be an efficient and promising classifier for large scale data sets processing.

**Conclusions:**

This paper extends the statistical framework of genomic data fusion based on MKL. Allowing non-sparse weights on the data sources is an attractive option in settings where we believe most data sources to be relevant to the problem at hand and want to avoid a "winner-takes-all" effect seen in *L*_∞ _MKL, which can be detrimental to the performance in prospective studies. The notion of optimizing *L*_2 _kernels can be straightforwardly extended to ranking, classification, regression, and clustering algorithms. To tackle the computational burden of MKL, this paper proposes several novel LSSVM based MKL algorithms. Systematic comparison on real data sets shows that LSSVM MKL has comparable performance as the conventional SVM MKL algorithms. Moreover, large scale numerical experiments indicate that when cast as semi-infinite programming, LSSVM MKL can be solved more efficiently than SVM MKL.

**Availability:**

The MATLAB code of algorithms implemented in this paper is downloadable from http://homes.esat.kuleuven.be/~sistawww/bioi/syu/l2lssvm.html.

## Background

In the era of information overflow, data mining and machine learning are indispensable tools to retrieve information and knowledge from data. The idea of incorporating several data sources in analysis may be beneficial by reducing the noise, as well as by improving statistical significance and leveraging the interactions and correlations between data sources to obtain more refined and higher-level information [1], which is known as *data fusion*. In bioinformatics, considerable effort has been devoted to *genomic data fusion*, which is an emerging topic pertaining to a lot of applications. At present, terabytes of data are generated by high-throughput techniques at an increasing rate. In data fusion, these terabytes are further multiplied by the number of data sources or the number of species. A statistical model describing this data is therefore not an easy matter. To tackle this challenge, it is rather effective to consider the data as being generated by a complex and unknown black box with the goal of finding a function or an algorithm that operates on an input to predict the output. About 15 years ago, Vapnik [2] introduced the support vector method which makes use of kernel functions. This method has offered plenty of opportunities to solve complicated problems but also brought lots of interdisciplinary challenges in statistics, optimization theory, and the applications therein [3].

Multiple kernel learning (MKL) has been pioneered by Lanckriet *et al*. [4] and Bach *et al*. [5] as an additive extension of single kernel SVM to incorporate multiple kernels in classification. It has also been applied as a statistical learning framework for genomic data fusion [6] and many other applications [7]. The essence of MKL, which is the additive extension of the dual problem, relies only on the kernel representation (kernel trick) while the heterogeneities of data sources are resolved by transforming different data structures (i.e., vectors, strings, trees, graphs, etc.) into kernel matrices. In the dual problem, these kernels are combined into a single kernel, moreover, the coefficients of the kernels are leveraged adaptively to optimize the algorithmic objective, known as *kernel fusion*. The notion of kernel fusion was originally proposed to solve classification problems in computational biology, but recent efforts have lead to analogous solutions for one class [7] and unsupervised learning problems (Yu *et al*.: Optimized data fusion for kernel K-means clustering, submitted). Currently, most of the existing MKL methods are based on the formulation proposed by Lanckriet *et al*. [4], which is clarified in our paper as the optimization of the infinity norm (*L*_∞_) of kernel fusion. Optimizing *L*_∞ _MKL in the dual problem corresponds to posing *L*_1 _regularization on the kernel coefficients in the primal problem. As known, *L*_1 _regularization is characterized by the sparseness of the kernel coefficients [8]. Thus, the solution obtained by *L*_∞ _MKL is also sparse, which assigns dominant coefficients to only one or two kernels. The sparseness is useful to distinguish relevant sources from a large number of irrelevant data sources. However, in biomedical applications, there are usually a small number of sources and most of these data sources are carefully selected and preprocessed. They thus often are directly relevant to the problem. In these cases, a sparse solution may be too selective to thoroughly combine the complementary information in the data sources. While the performance on benchmark data may be good, the selected sources may not be as strong on truly novel problems where the quality of the information is much lower. We may thus expect the performance of such solutions to degrade significantly on actual real-world applications. To address this problem, we propose a new kernel fusion scheme by optimizing the *L*_2_-norm of multiple kernels. The *L*_2 _MKL yields a non-sparse solution, which smoothly distributes the coefficients on multiple kernels and, at the same time, leverages the effects of kernels in the objective optimization. Empirical results show that the *L*_2_-norm kernel fusion can lead to a better performance in biomedical data fusion.

## Methods

### Acronyms

The symbols and notations used in this paper are defined in Table 1 (in the order of appearance).

**Table 1:** Acronyms

	ℝ^*N*^	the dual variable of SVM
*Q*	ℝ^*N *× *N*^	a semi-positive definite matrix
*C*	ℝ^*N*^	a convex set
Ω	ℝ^*N *× *N*^	a combination of multiple semi-positive definite matrices
*j*	ℕ	the index of kernel matrices
*p*	ℕ	the number of kernel matrices
*θ*	[0, 1]	coefficients of kernel matrices
*t*	[0, + ∞)	dummy variable in optimization problem
	ℝ^*p*^	
	ℝ^*p*^	
	ℝ^*D *^or ℝ^Φ^	the norm vector of the separating hyperplane
*ϕ*(·)	ℝ^*D *^→ ℝ^Φ^	the feature map
*i*	ℕ	the index of training samples
	ℝ^*D*^	the vector of the *i*-th training sample
*ρ*	ℝ	bias term in 1-SVM
*ν*	ℝ^+^	regularization term of 1-SVM
*ξ*_*i*_	ℝ	slack variable for the *i*-th training sample
*K*	ℝ^*N *× *N*^	kernel matrix
	ℝ^*D *^× ℝ^*D *^→ ℝ	kernel function,
	ℝ^*D*^	the vector of a test data sample
*y*_*i*_	-1 or +1	the class label of the *i*-th training sample
*Y*	ℝ^*N *× *N*^	the diagonal matrix of class labels *Y *= *diag*(*y*_1_, ..., *y*_*N*_)
*C*	ℝ^+^	the box constraint on dual variables of SVM
*b*	ℝ^+^	the bias term in SVM and LSSVM
	ℝ^*p*^	
*k*	ℕ	the number of classes
	ℝ^*p*^	
	ℝ^*p*^	variable vector in SIP problem
*u*	ℝ	dummy variable in SIP problem
*q*	ℕ	the index of class number in classification problem, *q *= 1, ..., *k*
*A*	ℝ^*N *× *N*^	
λ	ℝ^+^	the regularization parameter in LSSVM
*e*_*i*_	ℝ	the error term of the *i*-th sample in LSSVM
	ℝ^*N*^	the dual variable of LSSVM,
*ϵ*	ℝ^+^	precision value as the stopping criterion of SIP iteration
*τ*	ℕ	index parameter of SIP iterations
	ℝ^*p*^	

### Formal definition of the problem

We consider the problem of minimizing a quadratic cost of a real vector in function of  and a real positive semi-definite (PSD) matrix *Q*, given by(1)

where  denotes a convex set. Also, PSD implies that . We will show that many machine learning problems can be cast in form (1) with additional constraints on . In particular, if we restrict , the problem in (1) becomes a Rayleigh quotient and leads to the eigenvalue problem. Now we consider a convex parametric linear combination of a set of *p *PSD matrices *Q*_*j*_, given by:(2)

To bound the coefficients *θ*_*j*_, we restrict that, for example, ||*θ*_*j*_||_1 _= 1, and (1) can be equivalently rewritten as a min-max problem, given by(3)

To solve (3), we denote , the min-max problem can be formulated in a form of quadraticly constrained linear program (QCLP), given by(4)

The optimal solution  in (3) is obtained from the dual variable corresponding to the quadratic constraints in (4). The optimal t* is equivalent to the *Chebyshev *or *L*_∞_-norm of the vector of quadratic terms, given by:(5)

The *L*_∞_-norm is the upper bound w.r.t. the constraint  because(6)

Apparently, suppose the optimal  is given, optimizing the *L*_∞_-norm in (5) will pick the single term with the maximal value, and the optimal solution of the coefficients is more likely to be sparse. An alternative solution to (3) is to introduce a different constraint on the coefficients, for example, ||*θ*_*j*_||_2 _= 1. We thus propose a new extension of the problem in (1), given by(7)

This new extension is analogously solved as a QCLP problem with modified constraints, given by(8)

where . The proof that (8) is the solution of (7) is given in the following theorem.

**Theorem 0.1 ***The QCLP problem in (8) equivalently solves the problem in (7)*.

**Proof **Given two vectors {*x*_1_, ..., *x*_*p*_}, {*y*_1_, ..., *y*_*p*_}, *x*_*j*_, *y*_*j *_∈ ℝ, *j *= 1, ..., *p*, the Cauchy-Schwarz inequality states that(9)

with as equivalent form:(10)

Let us denote *x*_*j *_= *θ*_*j *_and , (10) becomes(11)

Since ||*θ*_*j*_||_2 _= 1, (11) is equivalent to(12)

Therefore, given , the additive term  is bounded by the *L*_2_-norm ||||_2_.

Moreover, it is easy to prove that when , the parametric combination reaches the upperbound and the equality holds. Optimizing this *L*_2_-norm results in a non-sparse solution in *θ*_*j*_. In order to distinguish this from the solution obtained by (3) and (4), we denote it as the *L*_2_-norm approach. It can also easily be seen (not shown here) that the *L*_1_-norm approach is simply averaging the quadratic terms with uniform coefficients.

The *L*_2_-norm bound is also generalizable to any positive real number *n *≥ 1, defined as *L*_*n*_-norm MKL. Recently, the similar topic is also investigated by [9] and a solution is proposed to solve the primal MKL problem. In this paper, we will show that our primal-dual interpretation of MKL is also extendable to the *n*-norm. Let us assume that  is regularized by the *L*_*m*_-norm as ||||_*m *_= 1, then the *L*_*m*_-norm extension of equation (7) is given by(13)

In the following theorem, we prove that (13) can be equivalently solved as a QCLP problem, given by(14)

where  and the constraint is in *L*_*n*_-norm, moreover, . The problem in (14) is convex and can be solved by cvx toolbox [10,11].

**Theorem 0.2 ***If the coefficient vector **is regularized by a L*_*m*_*-norm in (13), the problem can be solved as a convex programming problem in (14) with L*_*n*_*-norm constraint. Moreover*, .

**Proof **We generalize the Cauchy-Schwarz inequality to Hölder's inequality. Let *m*, *n *> 1 be two numbers that satisfy . Then(15)

Let us denote *x*_*j *_= *θ*_*j *_and , (2) becomes(16)

Since ||||_*m *_= 1, therefore the term  can be omitted in the equation, so (3) is equivalent to(17)

Due to the condition that , so , we prove that with the *L*_*m*_-norm constraint posed on , the additive multiple kernel term  is bounded by the *L*_*n*_-norm of the vector . Moreover, we have .

In this section, we have explained the *L*_∞_, *L*_1_, *L*_2_, and *L*_*n*_-norm approaches to extend the basic problem in (1) to multiple matrices *Q*_*j*_. These approaches differed mainly on the constraints applied on the coefficients. To clarify the difference of notations used in this paper with the common interpretations of *L*_1 _and *L*_2 _regularization on , we illustrate the mapping of our *L*_∞_, *L*_1_, *L*_2_, and *L*_*n *_notations between the common interpretations of coefficient regularization. As shown in Table 2, the notations used in this paper are interpreted in the dual space and are equivalent to regularization of kernel coefficients in the primal space. The advantage of dual space interpretation is that we can easily extend the analogue solution to various machine learning algorithms. To keep the discussion concise, we will from now on mainly focus on comparing the *L*_∞_, *L*_1 _and *L*_2 _in the dual problems and present the solutions in the dual space.

**Table 2:** The notation used in this paper is based on the dual problem and can be linked to a equivalent notation in the primal problem

	primal problem	dual problem
variable	***θ***_***j***_	
**L**_∞_		
**L**_**1**_		
**L**_**2**_		
**L**_**1.5**_		
**L**_**1.3333**_		
**L**_**1.25**_		
**L**_**1.2**_		
**L**_**1.1667**_		

Next, we will investigate several concrete kernel fusion algorithms and will propose the corresponding *L*_2 _solutions.

### One class SVM kernel fusion for ranking

The primal problem of one class SVM (1-SVM) is defined by Tax and Duin [12] and Schölkopf *et al*. [13] as(18)

where  is the norm vector of the separating hyperplane,  are the training samples, *ν *is the regularization constant penalizing outliers in the training samples, *ϕ*(·) denotes the feature map, *ρ *is a bias term, *ξ*_*i *_are slack variables, and *N *is the number of training samples. Taking the conditions for optimality from the Lagrangian, one obtains the dual problem, given by:(19)

where *α*_*i *_are dual variables, *K *represents the kernel matrix obtained by the inner product between any pair of samples specified by a kernel function . To incorporate multiple kernels in (19), De Bie *et al*. proposed a solution [7] with the dual problem formulated as(20)

where *p *is the number of data sources and *K*_*j *_is the *j*-th kernel matrix. The formulation exactly corresponds to the *L*_∞ _solution of the problem defined in the previous section (the PSD constraint is implied in the kernel matrix) with additional constraints imposed on . The optimal coefficients *θ*_*j *_are used to combine multiple kernels as(21)

and the ranking function is given by(22)

where Ω_*N *_is the combined kernel of training data , *i *= 1, ..., *N*,  is the test data point to be ranked,  is the kernel function applied on test data and training data,  is the dual variable solved as (20). De Bie *et al*. applied the method in the application of disease gene prioritization, where multiple genomic data sources are combined to rank a large set of test genes using the 1-SVM model trained from a small set of training genes which are known to be relevant for certain diseases. The *L*_∞ _formulation in their approach yields a sparse solution when integrating genomic data sources (see Figure 2 of [7]). To avoid this disadvantage, they proposed a regularization method by restricting the minimal boundary on the kernel coefficients, notated as *θ*_*min*_, to ensure the minimal contribution of each genomic data source to be *θ*_*min*_/*p*. According to their experiments, the regularized solution performed best, being significantly better than the sparse integration and the average combination of kernels.

Instead of setting the ad hoc parameter *θ*_*min*_, one can also straightforwardly propose an *L*_2_-norm approach to solve the identical problem, given by(23)

where . The problem above is a QCLP problem and can be solved by conic optimization solvers such as Sedumi [14]. In (23), the first constraint represents a Lorentz cone and the second constraint corresponds to *p *number of rotated Lorentz cones (R cones). The optimal kernel coefficients *θ*_*j *_correspond to the dual variables of the R cones with ||*θ*||_2 _= 1. In this *L*_2_-norm approach, the integrated kernel Ω is combined by different  and the same scoring function as in (22) is applied on the different solutions of  and Ω.

### Support vector machine MKL for classification

The notion of MKL is originally proposed in a binary SVM classification, where the primal objective is given by(24)

where  are data samples, *ϕ*(·) is the feature map, *y*_*i *_are class labels, *C *> 0 is a positive regularization parameter, *ξ*_*i *_are slack variables,  is the norm vector of the separating hyperplane, and *b *is the bias. This problem is convex and can be solved as a dual problem, given by(25)

where  are the dual variables, *Y *= *diag*(*y*_1_, ..., *y*_*N*_), *K *is the kernel matrix, and *C *is the upperbound of the box constraint on the dual variables. To incorporate multiple kernels in (25), Lanckriet *et al*. [6,4] and Bach *et al*. [5] proposed a multiple kernel learning (MKL) problem as follows:(26)

where *p *is the number of kernels. (26) optimizes the *L*_∞_-norm of the set of kernel quadratic terms. Based on the previous discussions, the *L*_2_-norm solution is analogously given by(27)

where . Both formulations in (26) and (27) can be efficiently solved as second order cone programming (SOCP) problems by a conic optimization solver (i.e., Sedumi [14]) or as QCQP problems by a general QP solver (i.e., MOSEK [15]). It is also known that a binary MKL problem can also be formulated as Semi-definite Programming (SDP), as proposed by Lanckriet *et al*. [4] and Kim *et al*. [16]. However, in a multi-class problem, SDP problems are computationally prohibitive due to the presence of PSD constraints and can only be solved approximately by relaxation [17]. On the contrary, the QCLP and QCQP formulations of binary classification problems can be easily extended to a multi-class setting using the one-versus-all (1vsA) coding, i.e., solving the problem of *k *classes as *k *number of binary problems. Therefore, the *L*_∞ _multi-class SVM MKL is then formulated as(28)

The *L*_2 _multi-class SVM MKL is given by(29)

where

#### SIP formulation for SVM MKL on larger scale data

Unfortunately, the kernel fusion problem becomes challenging on large scale data because it may scale up in three dimensions: the number of data points, the number of classes, and the number of kernels. When these dimensions are all large, memory issues may arise as the kernel matrices need to be stored in memory. Though it is feasible to approximate the kernel matrices by a low rank decomposition (i.e., incomplete Cholesky decomposition) and to reduce the computational burden of conic optimization using these low rank matrices, conic problems involve a large amount of variables and constraints and it is usually less efficient than QCQP. Moreover, the precision of the low rank approximation relies on the assumption that the eigenvalues of kernel matrices decay rapidly, which may not always be true when the intrinsic dimensions of the kernels are large. To tackle the computational burden of MKL, Sonnenburg *et al*. reformulated the QP problem as semi-infinite programming (SIP) and approximated the QP solution using a bi-level strategy (wrapper method) [18]. The standard form of SIP is given by(30)

where the constraint functions in  can be either linear or quadratic and there are infinite number of them in ∀*t *∈ ϒ. To solve it, a *discretization *method is usually applied, which is briefly summarized as follows [19-21]:

1. Choose a finite subset  ⊂ ϒ.

2. Solve the convex programming problem(31)(32)

3. If the solution of 2 is not satisfactorily close to the original problem then choose a larger, but still finite subset  and repeat from Step 2.

The convergence of SIP and the accuracy of the discretization method have been extensively described (see [19-21]). As proposed by Sonnenburg *et al*. [18], the multi-class SVM MKL objective in (26) can be formulated as a SIP problem, given by(33)

The SIP problem above is solved as a bi-level algorithm for which the pseudo code is presented in Algorithm 1 in the Appendix. In each loop *τ*, Step 1 optimizes  and *u*^(*τ*) ^for a restricted subset of constraints as a linear programming. Step 3 is an SVM problem with a single kernel and generates a new . If  is not satisfied by the current  and *u*^(*τ*)^, it will be added successively to step 1 until all constraints are satisfied. The starting points  are randomly initialized and SIP always converges to a identical result.

Algorithm 1 is also applicable to the *L*_2_-norm situation of SVM MKL, whereas the non-convex constraint  in Step 1 needs to be relaxed as , and the *f*_*j*_() term in (32) is modified as only containing the quadratic term. The SIP formulation for *L*_2_-norm SVM MKL is given by(34)

With these modifications, Step 1 of Algorithm 1 becomes a QCLP problem given by(35)

where  and  is a given value. Moreover, the PSD property of kernel matrices ensures that *A*_*j *_≥ 0, thus the optimal solution always satisfies .

In the SIP formulation, the SVM MKL is solved iteratively as two components. The first component is a single kernel SVM, which is solved more efficiently when the data scale is larger then thousands of data points (and smaller than ten thousands) and, requires much less memory than the QP formulation. The second component is a small scale problem, which is a linear problem in *L*_∞ _case and a QCLP problem in the *L*_2 _approach. As shown, the complexity of the SIP based SVM MKL is mainly determined by the burden of a single kernel SVM multiplied by the number of iterations. This has inspired us to adopt more efficient single SVM learning algorithms to further improve the efficiency. The least squares support vector machines (LSSVM) [22] is known for its simple differentiable cost function, the equality constraints in the separating hyperplane and its solution based on linear equations, which is preferable for large scaler problems. Next, we will investigate the MKL solutions issue using LSSVM formulations.

### Least squares SVM MKL for classification

In LSSVM, the primal problem is given by [22](36)

where most of the variables are defined in a similar way as in (24). The main difference is that the nonnegative slack variable *ξ *is replaced by a squared error term  and the inequality constraints are modified as equality ones. Taking the conditions for optimality from the Lagrangian, eliminating , defining  = [*y*_1_, ..., *y*_*N*_] and *Y *= *diag*(*y*_1_, ..., *y*_*N*_), one obtains the following linear system [22]:(37)

where  are unconstrained dual variables. Without the loss of generality, we denote  and rewrite (37) as(38)

In (38), we add an additional constraint as *Y*^-2 ^= *I *then the coefficient becomes a static value in the multi-class case. In 1vsA coding, (37) requires to solve *k *number of linear problems whereas in (38), the coefficient matrix is only factorized once such that the solution of  w.r.t. the multi-class label vectors  is very efficient to obtain. The constraint *Y*^-2 ^= *I *can be simply satisfied by assuming the class labels to be -1 and +1. Thus, from now on, we assume *Y*^-2 ^= *I *in the following discussion.

To incorporate multiple kernels in LSSVM classification, the *L*_∞_-norm approach is a QP problem, given by (assuming *Y*^-2 ^= *I*)(39)

The *L*_2_-norm approach is analogously formulated as(40)

where . The λ parameter regularizes the squared error term in the primal objective in (36) and the quadratic term  in the dual problem. Usually, the optimal λ needs to be selected empirically by cross-validation. In the kernel fusion of LSSVM, we can alternatively transform the effect of regularization as an identity kernel matrix in , where *θ*_*p *+ 1 _= 1/λ. Then the MKL problem of combining *p *kernels is equivalent to combining *p *+ 1 kernels where the last kernel is an identity matrix with the optimal coefficient corresponding to the λ value. This method has been mentioned by Lanckriet *et al*. to tackle the estimation of the regularization parameter in the soft margin SVM [4]. It has also been used by Ye *et al*. to jointly estimate the optimal kernel for discriminant analysis [17]. Saving the effort of validating λ may significantly reduce the model selection cost in complicated learning problems. By this transformation, the objective of LSSVM MKL becomes similar to that of SVM MKL with the main difference that the dual variables are unconstrained. Though (39) and (40) can in principle both be solved as QP problems by a conic solver or a QP solver, the efficiency of a linear solution of the LSSVM is lost. Fortunately, in a SIP formulation, the LSSVM MKL can be decomposed into iterations of the master problem of single kernel LSSVM learning, which is an unconstrained QP problem, and a coefficient optimization problem with very small scale.

#### SIP formulation for LSSVM SVM MKL on larger scale data

The *L*_∞_-norm approach of multi-class LSSVM MKL is formulated as(41)

In the formulation above, *K*_*j *_represents the *j *--th kernel matrix in a set of *p *+ 1 kernels with the *p *+ 1-th kernel being the identity matrix. The *L*_2_-norm LSSVM MKL is formulated as(42)

The pseudocode of *L*_∞ _-norm and *L*_2_-norm LSSVM MKL is presented in Algorithm 2 in the Appendix. In *L*_∞ _approach, Step 1 optimizes  as a linear programming. In *L*_2 _approach, Step 1 optimizes  as a QCLP problem. Since the regularization coefficient is automatically estimated as *θ*_*p *+ 1_, Step 3 simplifies to a linear problem as(43)

where .

### Summary of algorithms

As discussed, the dual *L*_2 _MKL solution can be extended to many machine learning problems. In principle, all MKL algorithms can be formulated in *L*_∞_, *L*_1_, and *L*_2 _forms and lead to different solutions. To validate the proposed approach, we implemented and compared 20 algorithms on various data sets. The summary of all implemented algorithms is presented in Table 3. These algorithms combine *L*_*∞*_, *L*_1_, and *L*_2 _MKL with 1-SVM, SVM, and LSSVM. Moreover, to cope with imbalanced data in classification, we also extended Weighted SVM [23,24] and Weighted LSSVM [25,26] to their MKL formulations (presented in Additional file 1). Though we mainly focus on *L*_∞_, *L*_1_, and *L*_2 _MKL methods, we also implement the *L*_*n*_-norm MKL for 1-SVM, SVM, LS-SVM and Weighted SVM. These algorithms are applied on the four biomedical experimental data sets and the performance is reported in section 8 of Additional file 1. Moreover, the *L*_*n*_-norm algorithms are also available on the website of this paper.

**Table 3:** Summary of algorithms implemented in the paper

Algorithm Nr.	Formulation Nr.	Name	References	Formulation	Equations
1	1-A	1-SVM *L*_∞ _MKL	[7]	SOCP	(20)
1	1-B	1-SVM *L*_∞ _MKL	[7]	QCQP	(20)
2	2-A	1-SVM *L*_∞ _(0.5) MKL	[7]	SOCP	(20)
2	2-B	1-SVM *L*_∞ _(0.5) MKL	[7]	QCQP	(20)
3	3-A	1-SVM *L*_1 _MKL	[12,13]	SOCP	(19)
3	3-B	1-SVM *L*_1 _MKL	[12,13]	QCQP	(19)
4	4-A	1-SVM *L*_2 _MKL	novel	SOCP	(23)
5	5-B	SVM *L*_∞ _MKL	[4,6,5]	QCQP	(26)
5	5-C	SVM *L*_∞ _MKL	[18]	SIP	(33)
6	6-B	SVM *L*_∞ _(0.5) MKL	novel	QCQP	(26)
7	7-A	SVM *L*_1 _MKL	[2]	SOCP	(25)
7	7-B	SVM *L*_1 _MKL	[4]	QCQP	(25)
8	8-A	SVM *L*_2 _MKL	novel	SOCP	(27)
8	8-C	SVM *L*_2 _MKL	[40]	SIP	(34)
9	9-B	Weighted SVM *L*_∞ _MKL	novel	QCQP	Suppl. (3)
10	10-B	Weighted SVM *L*_∞ _(0.5) MKL	novel	QCQP	Suppl. (3)
11	11-B	Weighted SVM *L*_1 _MKL	[25]	QCQP	Suppl. (2)
12	12-A	Weighted SVM *L*_2 _MKL	novel	SOCP	Suppl. (4)
13	13-B	LSSVM *L*_∞ _MKL	[17]	QCQP	(39)
13	13-C	LSSVM *L*_∞ _MKL	[17]	SIP	(41)
14	14-B	LSSVM *L*_∞ _(0.5) MKL	novel	QCQP	(39)
15	15-D	LSSVM *L*_1 _MKL	[22]	linear	(38)
16	16-B	LSSVM *L*_2 _MKL	novel	SOCP	(40)
16	16-C	LSSVM *L*_2 _MKL	novel	SIP	(42)
17	17-B	Weighted LSSVM *L*_∞ _MKL	novel	QCQP	Suppl. (8)
18	18-B	Weighted LSSVM *L*_∞ _(0.5) MKL	novel	QCQP	Suppl. (8)
19	19-D	Weighted LSSVM *L*_1 _MKL	[25]	linear	Suppl. (6)
20	20-A	Weighted LSSVM *L*_2 _MKL	novel	SOCP	Suppl. (9)

Summary of algorithms implemented in the paper. Because a same algorithm can be solved via different formulations. The different formulation numbers correspond to a same algorithm number and represent multiple formulations. In total 20 different algorithms were implemented, which were solved through 28 different formulations. For an algorithm with different formulations, the solutions are identical and only differ by computational efficiency. Some algorithms have already been proposed in the literature as shown in the reference column. The novel algorithms and formulations proposed in this paper are labeled as "novel".

### Experimental setup and data sets

The performance of the proposed *L*_2 _MKL method was systematically evaluated and compared on six real benchmark data sets. The computational efficiency was compared on two UCI data sets. On each data set, we compared the *L*_2 _method with the *L*_∞_, *L*_1 _and regularized *L*_∞ _MKL method. In the regularized *L*_∞_, we set the minimal boundary of kernel coefficients *θ*_*min *_to 0.5, denoted as *L*_∞ _(0.5). We also compared the three different optimization formulations SOCP, QCQP and SIP on the UCI data sets. The experiments were categorized in five groups as summarized in Table 4.

**Table 4:** Summary of data sets and algorithms used in five experiments

Nr.	Data Set	Problem	Samples	Classes	Algorihtms	Evaluation
1	disease relevant genes	ranking	620	1	1-4	LOO AUC
2	prostate cancer genes	ranking	9	1	1-4	AUC
3	rectal cancer patients	classification	36	2	5-8,13-16	LOO AUC
4	endometrial disease	classification	339	2	5-8,13-16	3-fold AUC
	miscarriage	classification	2356	2	5-8,13-16	3-fold AUC
	pregnancy	classification	856	2	9-12,17-20	3-fold AUC
5	UCI pen digit and optical digit	classification	1000-3000	10	1A,1B,5B,5C,13B,13C	CPU time

#### Experiment 1

In the first experiment, we demonstrated a disease gene prioritization application to compare the performance of optimizing different norms in MKL. The computational definition of gene prioritization is mentioned in our earlier work [7,27,28]. In this paper, we applied four 1-SVM MKL algorithms to combine kernels derived from 9 heterogeneous genomic sources (shown in section 1 of Additional file 1) to prioritize 620 genes that are annotated to be relevant for 29 diseases in OMIM. The performance was evaluated by leave-one-out (LOO) validation: for each disease which contains *K *relevant genes, one gene, termed the "defector" gene, was removed from the set of training genes and added to 99 randomly selected test genes (test set). We used the remaining *K *- 1 genes (training set) to build our prioritization model. Then, we prioritized the test set of 100 genes with the trained model and determined the rank of that defector gene in test data. The prioritization function in (22) scored the relevant genes higher and others lower, thus, by labeling the "defector" gene as class "+1" and the random candidate genes as class "-1", we plotted the Receiver Operating Characteristic (ROC) curves to compare different models using the error of AUC (one minus the area under the ROC curve).

The kernels of data sources were all constructed using linear functions except the sequence data that was transformed into a kernel using a 2-mer string kernel function [29] (details in section 1 of Additional file 1). In total 9 kernels were combined in this experiment. The regularization parameter *ν *in 1-SVM was set to 0.5 for all comparing algorithms. Since there was no hyper-parameter needed to be tuned in LOO validation, we reported the LOO results as the performance of generalization. For each disease relevant gene, the 99 test genes were randomly selected in each LOO validation run from the whole human protein-coding genome. We repeated the experiment 20 times and the mean value and standard deviation were used for comparison.

#### Experiment 2

In the second experiment we used the same data sources and kernel matrices as in the previous experiment to prioritize 9 prostate cancer genes recently discovered by Eeles *et al*. [30], Thomas *et al*. [31] and Gudmundsson *et al*. [32]. A training set of 14 known prostate cancer genes was compiled from the reference database OMIM including only the discoveries prior to January 2008. This training set was then used to train the prioritization model. For each novel prostate cancer gene, the test set contained the newly discovered gene plus its 99 closest neighbors on the chromosome. Besides the error of AUC, we also compared the ranking position of the novel prostate cancer gene among its 99 closet neighboring genes. Moreover, we compared the MKL results with the ones obtained via the Endeavour application.

#### Experiment 3

The third experiment is taken from the work of Daemen *et al*. about the kernel-based integration of genome-wide data for clinical decision support in cancer diagnosis [33]. Thirty-six patients with rectal cancer were treated by combination of cetuximab, capecitabine and external beam radiotherapy and their tissue and plasma samples were gathered at three time points: before treatment (*T*_0_); at the early therapy treatment (*T*_1_) and at the moment of surgery (*T*_2_). The tissue samples were hybridized to gene chip arrays and after processing, the expression was reduced to 6,913 genes. Ninety-six proteins known to be involved in cancer were measured in the plasma samples, and the ones that had absolute values above the detection limit in less than 20% of the samples were excluded for each time point separately. This resulted in the exclusion of six proteins at *T*_0 _and four at *T*_1_. "Responders" were distinguished from "non-responders" according to the pathologic lymph node stage at surgery (pN-STAGE). The "responder" class contains 22 patients with no lymph node found at surgery whereas the "non-responder" class contains 14 patients with at least 1 regional lymph node. Only the two array-expression data sets (MA) measured at *T*_0 _and *T*_1 _and the two proteomics data sets (PT) measured at *T*_0 _and *T*_1 _were used to predict the outcome of cancer at surgery.

Similar to the original method applied on the data [33], we used R BioConductor DEDS as feature selection techniques for microarray data and the Wilcoxon rank sum test for proteomics data. The statistical feature selection procedure was independent to the classification procedure, however, the performance varied widely with the number of selected genes and proteins. We considered the relevance of features (genes and proteins) as prior knowledge and systematically evaluated the performance using multiple numbers of genes and proteins. According to the ranking of statistical feature selection, we gradually increased the number of genes and proteins from 11 to 36, and combined the linear kernels constructed by these features. The performance was evaluated by LOO method, where the reason was two folded: firstly, the number of samples was small (36 patients); secondly, the kernels were all constructed with a linear function. Moreover, in LSSVM classification we proposed the strategy to estimate the regularization parameter λ in kernel fusion. Therefore, no hyperparameter was needed to be tuned so we reported the LOO validation result as the performance of generalization.

#### Experiment 4

Our fourth experiment considered three clinical data sets. These three data sets were derived from different clinical studies and were used by Daemen and De Moor [34] as validation data for clinical kernel function development. Data set I contains clinical information on 402 patients with an endometrial disease who underwent an echographic examination and color Droppler [35]. The patients are divided into two groups according to their histology: malignant (hyperplasia, polyp, myoma, and carcinoma) versus benign (proliferative endometrium, secretory endometrium, atrophia). After excluding patients with incomplete data, the data contains 339 patients of which 163 malignant and 176 benign. Data set II comes from a prospective observational study of 1828 women undergoing transvaginal sonography before 12 weeks gestation, resulting in data for 2356 pregnancies of which 1458 normal at week 12 and 898 miscarriages during the first trimester [36]. Data set III contains data on 1003 pregnancies of unknown location (PUL) [37]. Within the PUL group, there are four clinical outcomes: a failing PUL, an intrauterine pregnancy (IUP), an ectopic pregnancy (EP) or a persisting PUL. Because persisting PULs are rare (18 cases in the data set), they were excluded, as well as pregnancies with missing data. The final data set consists of 856 PULs among which 460 failing PULs, 330 IUPs, and 66 EPs. As the most important diagnostic problem is the correct classification of the EPs versus non-EPs [38], the data was divided as 790 non-EPs and 66 EPs. To simulate a problem of combining multiple sources, for each data we created eight kernels and combined them using MKL algorithms for classification. The eight kernels included one linear kernel, three RBF kernels, three polynomial kernels and a clinical kernel. The kernel width of the first RBF kernel is selected by empirical rules as four times the average covariance of all the samples, the second and the third kernel widths were respectively six and eight times the average covariance. The degrees of the three polynomial kernels were set to 2, 3, and 4 respectively. The bias term of polynomial kernels was set to 1. The clinical kernels were constructed as proposed by Daemen and De Moor [33]. All the kernel functions are explained in section 3 of Additional file 1. We noticed that the class labels of the pregnancy data were quite imbalanced (790 non-EPs and 66 EPs). In literature, the class imbalanced problem can be tackled by modifying the cost of different classes in the objective function of SVM. Therefore, we applied weighted SVM MKL and weighted LSSVM MKL on the imbalanced pregnancy data. For the other two data sets, we compared the performance of SVM MKL and LSSVM MKL with different norms.

The performance of classification was benchmarked using 3-fold cross validation. Each data set was randomly and equally divided into 3 parts. As introduced in the Methods section, when combining multiple pre-constructed kernels in LSSVM based algorithms, the regularization parameter λ can be jointly estimated as the coefficient of identity matrix. In this case we don't need to optimize any hyper-parameter in the LSSVM. In the estimation approach of LSSVM and all approaches of SVM, we therefore could use both training and validation data to train the classifier, and test data to evaluate the performance. The evaluation was repeated three times, so each part was used once as test data. The average performance was reported as the evaluation of one repetition. In the standard validation approach of LSSVM, each dataset was partitioned randomly into three parts for training, validation and testing. The classifier was trained on the training data and the hyper-parameter λ was tuned on the validation data. When tuning the λ, its values were sampled uniformly on the log scale from 2^-10 ^to 2^10^. Then, at optimal λ, the classifier was retrained on the combined training and validation set and the resulting model is tested on the testing set. Obviously, the estimation approach is more efficient than the validation approach because the former approach only requires one training process whereas the latter needs to perform 22 times an additional training (21 λ values plus the model retraining). The performance of these two approaches was also investigated in this experiment.

#### Experiment 5

As introduced in the Methods section, a same MKL problem can be formulated as different optimization problems such as SOCP, QCQP, and SIP. The accuracy of the discretization method for solving SIP is mainly determined by the tolerance value *ε *predefined in the stopping criterion. In our implementation, *ε *was set to 5 × 10^-4^. These different formulations yield the same result but mainly differ on computational efficiency. In the fifth experiment we compared the efficiency of these optimization techniques on two large scale UCI data sets. The two data sets are digit recognition data for pen based handwriting recognition and optical based digit recognition. Both data sets contain more than 6000 data samples thus they were used as real large scale data sets to evaluate the computational efficiency. In our implementation, the optimization problems were solved by Sedumi [14], MOSEK [15] and the Matlab optimization toolbox. All the numerical experiments were carried on a dual Opteron 250 Unix system with 16 G memory and the computational efficiency was evaluated by the CPU time (in seconds).

## Results

### Experiment 1: disease relevant gene prioritization by genomic data fusion

**Figure 1:**
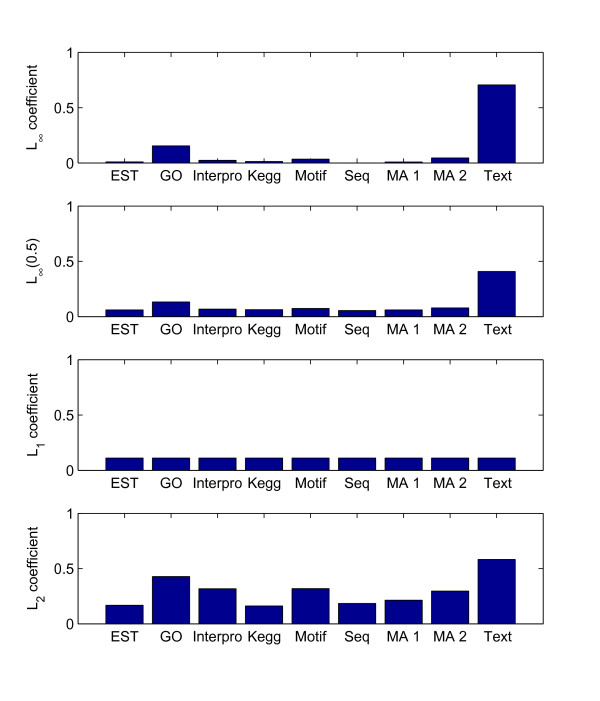
**Optimal kernel coefficients for disease gene prioritization**. Optimal kernel coefficients assigned on genomic data sources in disease gene prioritization. For each method, the average coefficients of 20 repetitions are shown. The three most important data sources ranked by *L*_∞ _are Text, GO, and Motif. The coefficients on other six sources are almost zero. The *L*_2 _method shows the same ranking on these three best data sources as *L*_∞_, moreover, it also shows ranking for other six sources. Thus, as another advantage of *L*_2 _method, it provides more refined ranking of data sources than *L*_∞ _method in data integration.

In the first experiment, the *L*_2 _1-SVM MKL algorithm performed the best (Error 0.0780). As shown in Table 5, the *L*_∞ _and *L*_1 _approaches all performed significantly worse than the *L*_2 _approach. For example, in the current experiment, when setting the minimal boundary of the kernel coefficients to 0.5, each data source was ensured to have a minimal contribution in integration, thereby improving the *L*_∞ _performance from 0.0923 to 0.0806, although still lower than *L*_2_. In Figure 1 we illustrate the optimal kernel coefficients of different approaches. As shown, the *L*_∞ _method assigned dominant coefficients to Text mining and Gene Ontology data, whereas other data sources were almost discarded from integration. In contrast, the *L*_2 _approach evenly distributed the coefficients over all data sources and thoroughly combined them in integration. When combining multiple kernels, sparse coefficients combine the model only with one or two kernels, making the combined model fragile with respect to the uncertainty and novelty. In real problems, the relevance of a new gene to a certain disease may not have been investigated thus a model solely based on Text and GO annotation is less reliable. *L*_2 _based integration evenly combines multiple genomic data sources. In this experiment, the *L*_2 _approach showed the same effect as the regularized *L*_∞ _by setting some minimal boundaries on kernel coefficients. However, in the regularized *L*_∞_, the minimal boundary *θ*_*min *_usually is predefined according to the "rule of thumb". The main advantage of the *L*_2 _approach is that the *θ*_*min *_values are determined automatically for different kernels and the performance is shown to be better with the manually selected values.

**Table 5:** Results of experiment 1: prioritization of 620 disease relevant genes by genomic data fusion

	Error of AUC (mean)	Error of AUC (std.)	p-value	corr	corr	corr	corr
*L*_*∞*_	0.0923	0.0035	2.98 · 10^-17^	-	0.94	0.66	0.82
*L*_*∞*_(0.5)	0.0806	0.0033	2.66 · 10^-06^	0.94	-	0.82	0.92
*L*_1_	0.0908	0.0042	1.92 · 10^-16^	0.66	0.82	-	0.90
*L*_2_	**0.0780**	0.0034	-	0.82	0.92	0.90	-

Results of experiment 1: disease relevant gene prioritization by genomic data fusion. The error of AUC values is evaluated by LOO validation in 20 random repetitions. The best performance (*L*_2_) is shown in bold. The p-values are compared with the best performance using a paired t-test. As shown, the *L*_2 _method is significantly better than other methods. The paired Spearman correlation scores compare similarities of rankings obtained by different approaches when compared with the target rankings (denoted as -). Higher Spearman correlation values mean that the two ranking results are much similar.

### Experiment 2: Prioritization of recently discovered prostate cancer genes by genomic data fusion

In the second experiment, recently discovered prostate cancer genes were prioritized using the same data sources and algorithms as in the first experiment. As shown in Table 6, the *L*_2 _method significantly outperformed other methods on prioritization of gene CDH23, and JAZF1. For 5 other genes (CPNE, EHBP1, MSMB, KLK3, IL16), the performance of the *L*_2 _method was comparable to the best result. In section 4 of Additional file 1, we also presented the optimal kernel coefficients and the prioritization results for individual sources. As shown in Additional file 1, the *L*_∞ _algorithm assigned most of the coefficients to Text and Microarray data. Text data performs well in the prioritization of known disease genes, however, does not always work the best for newly discovered genes. This experiment demonstrates that when prioritizing novel prostate cancer relevant genes, the *L*_2 _MKL approach evenly optimized the kernel coefficients to combine heterogeneous genomic sources and its performance was significantly better than the *L*_∞ _method. Moreover, we also compared the kernel based data fusion approach with the Endeavour gene prioritization software: for 6 genes the MKL approach performed significantly better than Endeavour.

**Table 6:** Results of experiment 2: prioritization of prostate cancer genes by genomic data fusion

Name	Ensemble id	References	***L***_∞_	***L***_∞_**(0.5)**	***L***_**1**_	***L***_**2**_	Endeavour
CPNE	ENSG00000085719	Thomas *et al*.	0.3030	0.2323	**0.1010**	*0.1212*	-
			31/100	24/100	**11/100**	*13/100*	70/100

CDH23	ENSG00000107736	Thomas *et al*.	0.0606	0.0303	*0.0202*	**0.0101**	-
			7/100	4/100	*3/100*	**2/100**	78/100

EHBP1	ENSG00000115504	Gudmundsson *et al*.	0.5354	0.5152	**0.3434**	*0.3939*	-
			54/100	52/100	**35/100**	*40/100*	57/100

MSMB	ENSG00000138294	Eeles *et al*.	**0.0202**	**0.0202**	0.0505	*0.0303*	-
		Thomas *et al*.	**3/100**	**3/100**	6/100	*4/100*	69/100

KLK3	ENSG00000142515	Eeles *et al*.	0.3434	0.3535	*0.2929*	*0.2929*	-
			35/100	36/100	*30/100*	*30/100*	**28/100**

JAZF1	ENSG00000153814	Thomas *et al*.	*0.0505*	**0.0202**	**0.0202**	**0.0202**	-
			*6/100*	**3/100**	**3/100**	**3/100**	7/100

LMTK2	ENSG00000164715	Eeles *et al*.	*0.3131*	0.4646	0.8081	0.7677	-
			*32/100*	47/100	81/100	77/100	**31/100**

IL16	ENSG00000172349	Thomas *et al*.	**0**	*0.0101*	0.0303	*0.0101*	-
			**1/100**	*2/100*	4/100	*2/100*	72/100

CTBP2	ENSG00000175029	Thomas *et al*.	*0.8283*	0.5758	*0.6364*	0.6869	-
			*83/100*	58/100	*64/100*	69/100	**38/100**

Results of experiment 2: prioritization of prostate cancer genes by genomic data fusion. For each novel prostate cancer gene, the first row shows the error of AUC values and the second row lists the ranking position of the prostate cancer gene among its 99 closet neighboring genes.

### Experiment 3: Clinical decision support by integrating microarray and proteomics data

**Figure 2:**
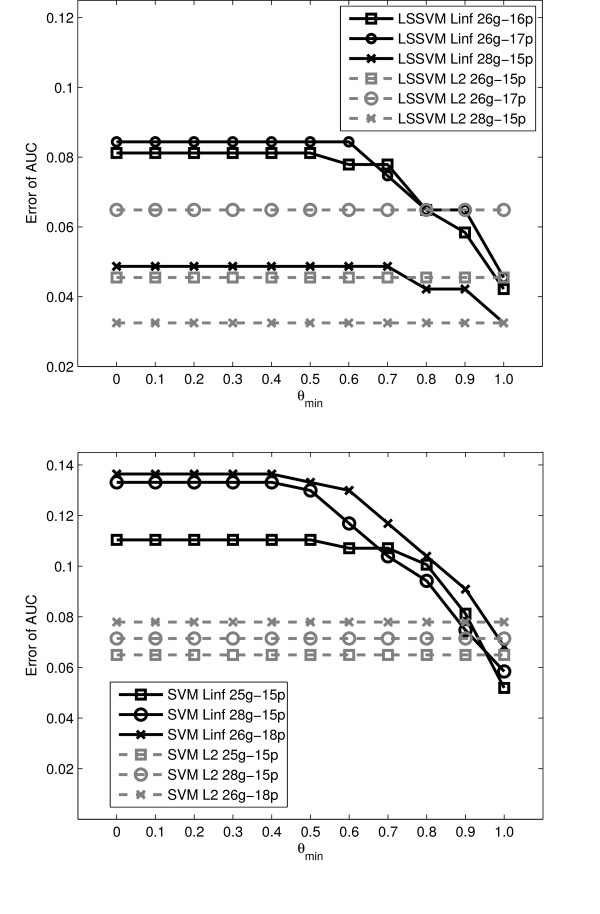
**The effect of *θ*_*min *_on LSSVM MKL and SVM MKL classifier in rectal cancer diagnosis**. The effect of *θ*_*min *_in LSSVM MKL and SVM MKL classifiers for rectal cancer diagnosis. Figure on the top: the performance of LSSVM MKL. Figure on the bottom: the performance of SVM MKL. In each figure we compare three feature selection results. The performance of *L*_2 _MKL is shown as dashed lines.

One of the main contributions of this paper is that the *L*_2 _MKL notion can be applied on various machine learning problems. The first two experiments demonstrated a ranking problem using 1-SVM MKL to prioritize disease relevant genes. In the third experiment we optimized the *L*_∞_, *L*_1_, and *L*_2 _-norm in SVM MKL and LSSVM MKL classifiers to support the diagnosis of patients according to their lymph node stage in rectal cancer development. The performance of the classifiers greatly depended on the selected features, therefore, for each classifier we compared 25 feature selection results (as a grid of 5 numbers of genes multiplied by 5 numbers of proteins). As shown in Table 7, the best performance was obtained with LSSVM *L*_1 _(error of AUC = 0.0325) using 25 genes and 15 proteins. The *L*_2 _LSSVM MKL classifier was also promising because its performance was comparable to the best result. In particular, for the two compared classifiers (LSSVM and SVM), the *L*_1 _and *L*_2 _approaches significantly outperformed the *L*_∞ _approach. We also tried to regularize the kernel coefficients in *L*_∞ _MKL using different *θ*_*min *_values. Nine different *θ*_*min *_were tried uniformly from 0.1 to 0.9 and the changes in performance is shown in Figure 2. As shown, increasing the *θ*_*min *_value steadily improves the performance of LSSVM MKL and SVM MKL on the rectal cancer data sets. However, determining the optimal *θ*_*min *_was a non-trivial issue. When *θ*_*min *_was smaller than 0.6, the performance of LSSVM MKL *L*_∞ _remained unchanged, meaning that the "rule of thumb" value 0.5 used in experiment 1 is not valid here. In comparison, when using the *L*_2 _based MKL classifiers, there is no need to specify *θ*_*min *_and the performance is still comparable to the best performance obtained with regularized *L*_∞ _MKL.

**Table 7:** Results of experiment 3: classification of patients in rectal cancer clinical decision using microarray and proteomics data sets

	**LSSVM *L*_∞_**					**SVM *L*_∞_**				
		
	**14 p**	**15 p**	**16 p**	**17 p**	**18 p**	**14 p**	**15 p**	**16 p**	**17 p**	**18 p**
	
24 g	0.0584	0.0519	*0.0747 *	0.0812	0.0812	0.1331	0.1331	0.1331	0.1331	0.1364
25 g	*0.0390*	*0.0390*	0.0519	0.0617	0.0649	0.1136	0.1104	0.1234	0.1201	0.1234
26 g	0.0487	0.0487	0.0812	0.0844	0.0877	0.1266	0.1136	0.1234	0.1299	0.1364
27 g	0.0617	0.0649	0.0812	0.0877	0.0942	0.1429	0.1364	0.1364	0.1331	0.1461
28 g	0.0552	0.0487	0.0617	0.0747	0.0714	0.1429	0.1331	0.1331	0.1364	0.1396
	
	LSSVM *L*_∞ _(0.5)					SVM *L*_∞ _(0.5)				
		
	14 p	15 p	16 p	17 p	18 p	14 p	15 p	16 p	17 p	18 p
24 g	0.0584	0.0519	*0.0747*	0.0812	0.0812	0.1266	0.1006	0.1266	0.1299	0.1331
25 g	*0.0390*	*0.0390*	0.0519	0.0617	0.0649	0.1136	0.1071	0.1234	0.1201	0.1234
26 g	0.0487	0.0487	0.0812	0.0844	0.0877	0.1136	0.1136	0.1201	0.1266	0.1331
27 g	0.0617	0.0649	0.0812	0.0877	0.0942	0.1364	0.1364	0.1364	0.1331	0.1461
28 g	0.0552	0.0487	0.0617	0.0747	0.0714	0.1299	0.1299	0.1299	0.1331	0.1364
	
	LSSVM *L*_1_					SVM *L*_1_				
		
	14 p	15 p	16 p	17 p	18 p	14 p	15 p	16 p	17 p	18 p
	
24 g	**0.0487**	**0.0487**	**0.0682**	**0.0682**	0.0747	0.0747	0.0584	0.0714	**0.0682**	0.0747
25 g	**0.0357**	**0.0325**	**0.0422**	**0.0455**	**0.0455**	0.0584	0.0519	0.0649	0.0714	0.0714
26 g	**0.0357**	**0.0357**	**0.0455**	**0.0455**	**0.0455**	0.0584	0.0519	0.0682	0.0682	0.0682
27 g	**0.0357**	**0.0357**	**0.0455**	**0.0487**	**0.0519**	0.0617	0.0584	0.0714	0.0682	0.0682
28 g	**0.0422**	**0.0325**	**0.0487**	**0.0487**	**0.0519**	0.0584	0.0584	0.0649	0.0649	0.0682
	
	LSSVM *L*_2_					SVM *L*_2_				
		
	14 p	15 p	16 p	17 p	18 p	14 p	15 p	16 p	17 p	18 p
	
24 g	*0.0552*	**0.0487**	*0.0747*	*0.0779*	**0.0714**	0.0909	0.0877	0.0974	0.0942	0.1006
25 g	*0.0390*	*0.0390*	*0.0487*	*0.0552*	*0.0552*	0.0747	0.0649	0.0812	0.0844	0.0844
26 g	*0.0390*	*0.0455*	*0.0552*	*0.0649*	*0.0649*	0.0747	0.0584	0.0812	0.0779	0.0779
27g	*0.0422*	*0.0487*	*0.0552*	*0.0584*	*0.0649*	0.0779	0.0812	0.0844	0.0812	0.0812
28 g	*0.0455*	**0.0325**	**0.0487**	*0.0584*	*0.0552*	0.0812	0.0714	0.0812	0.0779	0.0812

The table shows the error of AUC in patient classification using microarray and proteomics data. In LSSVM *L*_∞_, *L*_∞ _(0.5), and *L*_2_, the regularization parameter λ was estimated jointly as the kernel coefficient of an identity matrix. In LSSVM *L*_1_, λ was set to 1. In all SVM approaches, the *C *parameter of the box constraint was set to 1. In the table, the row and column labels represent the numbers of genes (g) and proteins (p) used to construct the kernels. The genes and proteins were ranked by feature selection techniques (see text). The AUC of LOO validation was evaluated without the bias term *b *(as the implicit bias approach) because its value varied by each left out sample. In this problem, considering the bias term decreased the AUC performance. The performance was compared among eight algorithms for the same number of genes and proteins, where the best values (the smallest Error of AUC) are represented in bold, the second best ones in italic. The best performance of all the feature selection results is underlined. The table presents the 25 best feature selection results of each method. The complete experimental results containing 26 different numbers of genes and 26 numbers of proteins is available at http://homes.esat.kuleuven.be/~sistawww/bioi/syu/l2lssvm.html.

**Figure 3:**
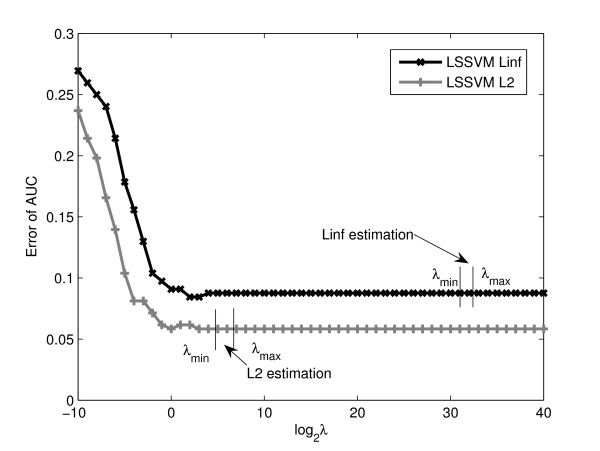
**Benchmark of various λ values in LSSVM MKL classifiers in rectal cancer diagnosis**. Benchmark of various λ values in LSSVM MKL classifiers for rectal cancer diagnosis. The four kernels were constructed using 27 gene features and 17 protein features (see text). For each fixed λ value, the error of AUC was evaluated by LOO validation. The maximal and minimal estimated λ in *L*_∞ _and *L*_2 _MKL are shown.

In LSSVM kernel fusion, we estimated the λ jointly as a coefficient assigned to an identity matrix. Since the number of samples is small in this experiment, the standard cross-validation approach to select the optimal λ on validation data was not tried. To investigate whether the estimated λ value is optimal, we set λ to 51 different values uniformly sampled on the *log*_2 _scale from -10 to 40. We compared the joint estimation result with the optimal classification performance among the sampled λ values. The joint estimation results were found as optimal for most of the results. An example is illustrated in Figure 3 for the integration of four kernels constructed by 27 gene features and 17 protein features. The coefficients estimated by the *L*_∞_-norm were almost 0 thus the λ values were very big. In contrast, the λ values estimated by the non-sparse *L*_2 _method were at reasonable scales.

### Experiment 4: Clinical decision support by integrating multiple kernels

**Figure 4:**
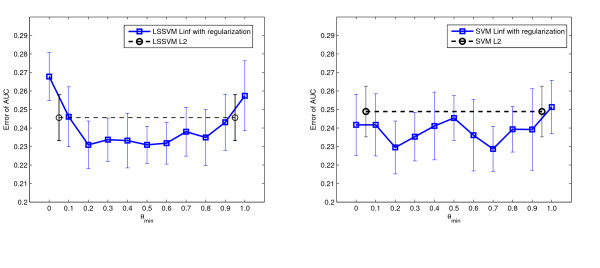
**The effect of *θ*_*min *_in LSSVM MKL and SVM MKL classifier on endometrial disease data set**. The effect of *θ*_*min *_in LSSVM MKL and SVM MKL classifiers on endometrial disease data set. Figure on the left: performance of the regularized LSSVM *L*_∞ _MKL with various *θ*_*min *_values. Figure on the right: performance of the regularized SVM *L*_∞ _MKL. The black dashed lines represent the performance of the *L*_2 _MKL classifiers. The error bars are standard deviations of 20 repetitions.

**Figure 5:**
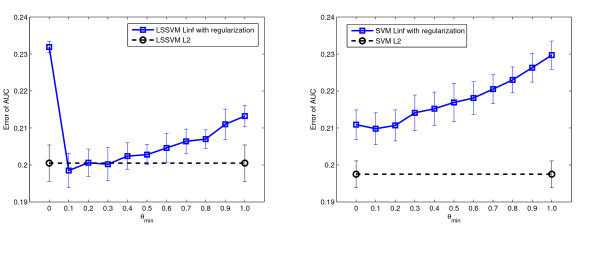
**The effect of *θ*_*min *_in LSSVM MKL and SVM MKL classifier on miscarriage data set**. The effect of *θ*_*min *_in LSSVM MKL and SVM MKL classifiers on miscarriage data set. Figure on the left: performance of the regularized LSSVM *L*_∞ _MKL with various *θ*_*min *_values. Figure on the right: performance of the regularized SVM *L*_∞ _MKL. The black dashed lines represent the performance of the *L*_2 _MKL classifiers. The error bars are standard deviations of 20 repetitions.

**Figure 6:**
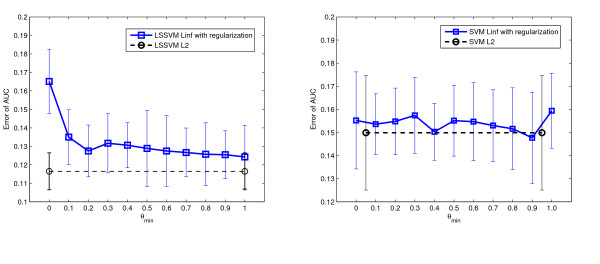
**The effect of *θ*_*min *_in weighted LSSVM MKL and weighted SVM MKL classifier on pregnancy data set**. The effect of *θ*_*min *_in LSSVM MKL and SVM MKL classifiers on pregnancy data set. Figure on the left: performance of the regularized LSSVM *L*_∞ _MKL with various *θ*_*min *_values. Figure on the right: performance of the regularized SVM *L*_∞ _MKL. The black dashed lines represent the performance of the *L*_2 _MKL classifiers. The error bars are standard deviations of 20 repetitions.

In the fourth experiment we validated the proposed approach on three clinical data sets containing more samples. On the endometrial and miscarriage data sets, we compared eight MKL algorithms with various norms. For the imbalanced pregnancy data set, we applied eight weighted MKL algorithms. The results are shown in Table 8, 9, and 10. On endometrial data, the difference of performance was rather small. Though the two *L*_2 _methods were not optimal, they were comparable to the best result. On miscarriage data, the *L*_2 _methods performed significantly better than comparing algorithms. On pregnancy data, the weighted *L*_2 _LSSVM MKL and weighted *L*_1 _LSSVM MKL performed significantly better than others. We also regularized the kernel coefficients using different *θ*_*min *_values on LSSVM *L*_∞ _and SVM *L*_∞ _MKL classifiers. The results are presented in Figure 4, Figure 5 and Figure 6. As shown, the optimal *θ*_*min *_value differs across data sets thus the "rule of thumb" value of 0.5 may not work for all the problems. For the endometrial and miscarriage data sets, the optimal *θ*_*min *_for both MKL classifiers is 0.2. For pregnancy data set, the optimal *θ*_*min *_value for LSSVM is 1 and for SVM 0.9. In comparison, on the miscarriage and pregnancy data set, the performance of the *L*_2 _algorithm is comparable or even much better than the best regularized *L*_∞ _algorithm. For the endometrial data set, though the optimal regularized *L*_*∞ *_LSSVM and SVM MKL classifiers outperform *L*_2 _classifiers, *L*_2 _methods still perform better than or as equal as the unregularized *L*_∞ _method.

**Table 8:** Results of experiment 4 data set I: classification of endometrial disease patients using multiple kernels derived from clinical data

Classifier	Mean - error of AUC	Std. - error of AUC	pvalue
**LSSVM ***L*_∞_** (0.5) MKL**	**0.2353**	**0.0133**	-
**SVM ***L*_∞_** (0.5) MKL**	**0.2388**	**0.0178**	0.4369
**SVM ***L*_∞_** MKL**	**0.2417**	**0.0165**	0.2483
LSSVM *L*_2 _MKL	0.2456	0.0124	0.0363
SVM *L*_2 _MKL	0.2489	0.0178	0.0130
SVM *L*_1 _MKL	0.2513	0.0144	0.0057
LSSVM *L*_1 _MKL	0.2574	0.0189	9.98 · 10^-5^
LSSVM *L*_∞ _MKL	0.2678	0.0130	1.53 · 10^-6^

Results of experiment 4 data set I: classification of endometrial disease patients using multiple kernels derived from clinical data. The classifier with the best performance is shown in bold. The p-values are compared with the best performance using a paired t-test. The performance of classifiers is sorted from high to low according to the p-values.

**Table 9:** Results of experiment 4 data set II: classification of miscarriage patients using multiple kernels derived from clinical data

Classifier	Mean - error of AUC	Std. - error of AUC	pvalue
**SVM ***L*_2 _**MKL**	**0.1975**	**0.0037**	-
**LSSVM ***L*_2 _**MKL**	**0.2002**	**0.0049**	0.0712
LSSVM *L*_∞ _(0.5) MKL	0.2027	0.0045	9.77 · 10^-4^
SVM *L*_∞ _MKL	0.2109	0.0040	9.55 · 10^-12^
SVM *L*_∞ _(0.5) MKL	0.2168	0.0040	1.79 · 10^-12^
LSSVM *L*_1 _MKL	0.2132	0.0029	1.11 · 10^-13^
SVM *L*_1 _MKL	0.2297	0.0038	1.10 · 10^-15^
LSSVM *L*_∞ _MKL	0.2319	0.0015	3.42 · 10^-21^

Results of experiment 4 data set II: classification of miscarriage patients using multiple kernels derived from clinical data. The classifier with the best performance is shown in bold. The p-values are compared with the best performance using a paired t-test. The performance of classifiers is sorted from high to low according to the p-values.

**Table 10:** Results of experiment 4 data set III: classification of PUL patients using multiple kernels derived from clinical data

Classifier	Mean - error of AUC	Std. - error of AUC	pvalue
**Weighted LSSVM ***L*_2 _**MKL**	**0.1165**	**0.0100**	-
**Weighted LSSVM ***L*_1 _**MKL**	**0.1243**	**0.0171**	0.0519
Weighted LSSVM *L*_∞ _(0.5) MKL	0.1290	0.0206	0.0169
Weighted SVM *L*_2 _MKL	0.1499	0.0248	4.79 · 10^-5^
Weighted SVM *L*_∞ _MKL	0.1552	0.0210	1.02 · 10-6
Weighted SVM *L*_∞ _(0.5)	0.1551	0.0153	3.87 · 10^-6^
Weighted SVM *L*_1 _MKL	0.1594	0.0162	2.29 · 10^-9^
Weighted LSSVM *L*_∞ _MKL	0.1651	0.0174	4.41 · 10^-10^

Results of experiment 4 data set II: classification of PUL patients using multiple kernels derived from clinical data. The classifier with the best performance is shown in bold. The p-values are compared with the best performance using a paired t-test. The performance of classifiers is sorted from high to low according to the p-values.

To investigate whether the combination of multiple kernels performs as well as the best individual kernel, we evaluated the performance of all the individual kernels in section 5 of Additional file 1. As shown, the clinical kernel proposed by Daemen and De Moor [33] has better quality than linear, RBF and polynomial kernels on endometrial and pregnancy data sets. For the miscarriage data set, the first RBF kernel has better quality than the other seven kernels. Despite the difference in individual kernels, the performance of MKL is comparable to the best individual kernel, demonstrating that MKL is also useful to combine candidate kernels derived from a single data set.

The effectiveness of MKL can also be justified by investigating the kernel coefficients optimized on all the data sets and classifiers. As shown in section 6 of Additional file 1, the kernel coefficients optimized by *L*_∞ _MKL algorithms were sparse whereas the *L*_2 _ones were more evenly assigned to different kernels. The best individual kernels of all data sets usually get dominant coefficient, explaining why the performance of MKL algorithms is comparable to the best individual kernels.

In this paper, the regularization parameter λ in LSSVM classifiers was jointly estimated in MKL. Since the clinical data sets contain a sufficient number of samples to select the λ by cross validation, we systematically compared the estimation approach with the standard validation approach to determine the λ values. As shown in Table 11, the estimation approach based on *L*_∞ _performed worse than the validation approach. This is probably because the estimated λ values are either very big or very small when the kernel coefficients were sparse. In contrast, the *L*_2 _based estimation approach yielded comparable performance as the validation approach. We also benchmarked the performance of LSSVM MKL classifiers using 21 different static λ values on the data sets and the results are shown in section 7 of Additional file 1. In real problems, to select the optimal λ value in LSSVM is a non-trivial issue and it is often optimized as a hyper-parameter on validation data. The main advantage of *L*_2 _MKL is that the estimation approach is more computational efficient than cross validation and yields a comparable performance.

**Table 11:** Comparison of the performance obtained by joint estimation of λ and standard cross-validation in LSSVM MKL

Data Set	Norm	Validation Approach	Estimation Approach
endometrial disease	*L*_∞_	0.2625 ± 0.0146	0.2678 ± 0.0130
	*L*_2_	0.2584 ± 0.0188	0.2456 ± 0.0124

miscarriage	*L*_∞_	0.1873 ± 0.0100	0.2319 ± 0.0015
	*L*_2_	0.1912 ± 0.0089	0.2002 ± 0.0049

pregnancy	*L*_∞_	0.1321 ± 0.0243	0.1651 ± 0.0173
	*L*_2_	0.1299 ± 0.0172	0.1165 ± 0.0100

Comparison of the performance obtained by joint estimation of λ and standard cross-validation using LSSVM MKL. As shown, the estimation approach based on *L*_2 _MKL is better than *L*_∞ _MKL. This is because when the kernel coefficients are sparse, the estimated regularization parameters λ are either very big or very small, which are usually not optimal values in LSSVM. In contrast, the λ values estimated by *L*_2 _method are at normal scale and often close to the optimal values.

### Experiment 5: Computational complexity and numerical experiments on large scale problems

#### Overview of the convexity and complexity

We concluded the convexity and the time complexity of all proposed methods in Table 12. All problems proposed in this paper are convex or can be transformed to a convex formulation by relaxation. The LSSVM SIP formulation has the lowest time complexity thus it is more preferable for large scale problems.

**Table 12:** Convexity and complexity of all methods

Method	convexity	complexity
1-SVM SOCP *L*_∞_, *L*_2_	convex	*O*((*p *+ *n*)^2^*n*^2.5^)
1-SVM QCQP *L*_∞_	convex	*O*(*pn*^3^)
SVM SOCP *L*_∞_, *L*_2_	convex	*O*((*p *+ *n*)^2^(*k + n*)^2.5^)
SVM QCQP *L*_∞_	convex	*O*(*pk*^2^*n*^2 ^+ *k*^3^*n*^3^)
SVM SIP *L*_∞_	convex	*O*(τ(*kn*^3 ^+ *p*^3^))
SVM SIP *L*_2_	relaxation	*O*(τ(*kn*^3 ^+ *p*^3^))
LSSVM SOCP *L*_∞_, *L*_2_	convex	*O*((*p *+ *n*)^2^(*k + n*)^2.5^)
LSSVM QCQP *L*_∞_, *L*_2_	convex	*O*(*pk*^2^*n*^2 ^+ *k*^3^*n*^3^)
LSSVM SIP *L*_∞_	convex	*O*(τ(*n*^2 ^+ *p*^3^))
LSSVM SIP *L*_2_	relaxation	*O*(τ(*n*^2 ^+ *p*^3^))

Convexity and complexity of all methods. *n *is the number of samples, *p *is the number of kernels, *k *is the number of classes, *τ *is the number of iterations in SIP. The complexity of LSSVM SIP depends on the algorithms used to solve the linear system. For the conjugate gradient method, the complexity is between *O*(*n*^1.5^) and *O*(*n*^2^) [22].

We verified the efficiency in numerical experiments, which adopts two UCI digit recognition data sets (pen-digit and optical digit) to compare the computational time of the proposed algorithms.

#### QP formulation is more efficient than SOCP

We investigated the efficiency of various formulations to solve the 1-SVM MKL. As mentioned, the problems presented in (15) can be solved either as QCLP or as SOCP. We applied Sedumi [14] to solve it as SOCP and MOSEK to solve it as QCLP and SOCP. We found that solving the QP by MOSEK was most efficient (142 seconds). In contrast, the MOSEK-SOCP method costed 2608 seconds and the Sedumi-SOCP method took 4500 seconds. This is probably because when transforming a QP to a SOCP, a large number of additional variables and constraints are involved, thus becoming more expensive to solve.

#### SIP formulation is more efficient than QCQP

**Figure 7:**
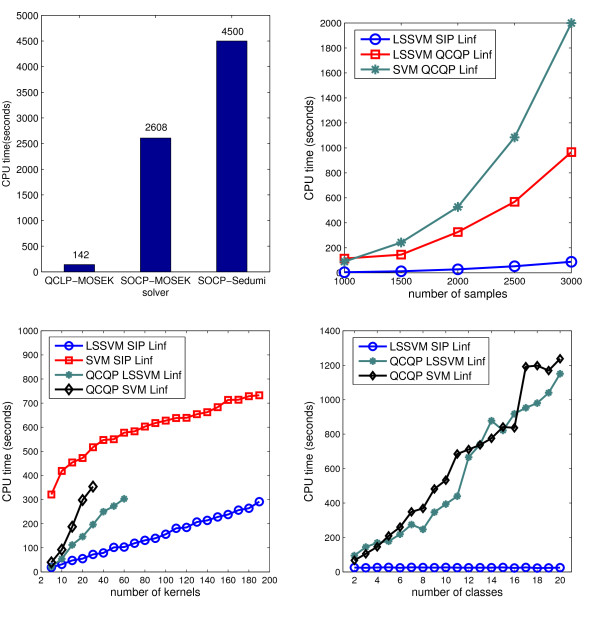
**Comparison of QP formulation and SIP formulation on large scale data**. Comparison of QP formulation and SIP formulation on large scale data. Figure on the top left: comparison of SOCP and QCQP formulations to solve 1-SVM MKL using two kernels. To simulate the ranking problem in 1-SVM, 3000 digit samples were retrieved as training data. Two kernels were constructed respectively for each data source using RBF kernel functions. The computational time was thus evaluated by combining the two 3000 × 3000 kernel matrices. Figure on the top right: comparison of SVM and LSSVM MKL on problems with increasing number of samples. The benchmark data set was made up of two linear kernels and labels in 10 digit classes. The number of data points was increased from 1000 to 3000. Figure on the bottom left: comparison of SVM and LSSVM MKL on problems with increasing number of kernels. The benchmark data set was constructed by 2000 samples labeled in 2 classes. We used different kernel widths to construct the RBF kernel matrices and increase the number of kernel matrices from 2 to 200. The QCQP formulations had memory issues when the number of kernels was larger than 60. Figure on the bottom right: comparison of SVM and LSSVM on problems with increasing number of classes. The benchmark data was made up of two linear kernel matrices and 2000 samples. The samples were equally and randomly divided into various number of classes. The class number gradually increased from 2 to 20.

To compare the computational time of solving MKL classifiers based on QP and SIP formulations, we scaled up the kernel fusion problem in three dimensions: the number of kernels, the number of classes and the number of samples. As shown in Figure 7, the SIP formulation of LSSVM MKL increases linearly with the number of samples and kernels, and is barely influenced by the number of classes. Solving the SIP based LSSVM MKL is significantly faster than solving SVM MKL because the former optimizes through iterations on a linear systems whereas the latter iterates over quadratic systems. For LSSVM MKL, the SIP formulation is also more preferable than the quadratic formulation. A quadratic system is a memory intensive problem and its complexity increases exponentially with the number of kernels and the number of samples in MKL. In contrast, the SIP formulation separates the problem into a series of linear systems, whose complexity is only determined by the number of samples and less affected by the number of kernels or classes. As shown in step 3 of Algorithm 5.2, the coefficient matrix of the linear system is a combined single kernel matrix and is constant with respect to multiple classes, thus it can be solved very efficiently. We have also compared the CPU time of *L*_∞ _and *L*_2 _LSSVM MKL on large data sets and their efficiency is very similar to each other.

## Discussion

In this paper we propose a new *L*_2 _MKL framework as the complement to the existing *L*_∞ _MKL method proposed by Lanckriet *et al*.. The *L*_2 _MKL is characterized by the non-sparse integration of multiple kernels to optimize the objective function of machine learning problems. On four real bioinformatics and biomedical applications, we systematically validated the performance through extensive analysis. The motivation for *L*_2 _MKL is as follows. In real biomedical applications, with a small number of sources that are believed to be truly informative, we would usually prefer a nonsparse set of coefficients because we would want to avoid that the dominant source (like text mining or Gene Ontology) gets a coefficient close to 1. The reason to avoid sparse coefficients is that there is a discrepancy between the experimental setup for performance evaluation and "real world" performance. The dominant source will work well on a benchmark because this is a controlled situation with known outcomes. We for example set up a set of already known genes for a given disease and want to demonstrate that our model can capture the available information to discriminate between a gene from this set and randomly selected genes (for example, in a cross-validation setup). Given that these genes are already known to be associated with the disease, this information will be present in sources like text mining or Gene Ontology in the gene prioritization problem. These sources can then identify these known genes with high confidence and should therefore be assigned a high weight. However, when trying to identify truly novel genes for the same disease, the relevance of the information available through such data sources will be much lower and we would like to avoid anyone data source to complete dominate the other. Given that setting up a benchmark requires knowledge of the association between a gene and a disease, this effect is hard to avoid. We can therefore expect that if we have a smoother solution that performs as well as the sparse solution on benchmark data, it is likely to perform better on real discoveries.

For the specific problem of gene prioritization, an effective way to address this problem is to setup a benchmark where information is "rolled back" a number of years (e.g., two years) prior to the discovery of the association between a gene and a disease (i.e., older information is used so that the information about the association between the gene and the disease is not yet contained in data sources like text mining or Gene Ontology). Given that the date at which the association was discovered is different for each gene, the setup of such benchmarks is notoriously difficult. In future work, we plan to address this problem by freezing available knowledge at a given data and then collecting novel discoveries and benchmarking against such discoveries in a fashion reminiscent of CASP (Critical Assessment of protein Structure Prediction) [39].

The technical merit of the proposed *L*_2 _MKL lay in the dual form of the learning problems. Though in the literature the issue of using different norms in MKL is recently investigated by Kloft *et al*. [40,9] and Kowalski *et al*. [41], their formulations are based on the primal problems. In our paper, the notion of the proposed *L*_2 _method is discussed in the dual space, which differs from regularizing the norm of coefficients term in the primal space. We have theoretically proven that optimizing the *L*_2 _regularization of kernel coefficients in the primal problem corresponds to solving the *L*_2_-norm of kernel components in the dual problem. Clarifying this dual solution enabled us to directly solve the *L*_2 _problem as a convex SOCP. Moreover, the dual solution can be extended to various other machine learning problems. In this paper we have shown the extensions of 1-SVM, SVM and LSSVM. As a matter of fact, the *L*_2 _dual solution can also be applied in kernel based clustering analysis and regression analysis for a wide range of applications. Another main contribution of our paper is the novel LSSVM *L*_2 _MKL proposed for classification problems. As known, when applying various machine learning techniques to solve real computational biological problems, the performance may depend on the data set and the experimental settings. When the performance evaluations of various methods are comparable, but with one method showing significant computational efficiency over other methods, this would be a "solid" advantage of this method. In this paper, we have shown that the LSSVM MKL classifier based on SIP formulation can be solved more efficiently than SVM MKL. Moreover, the performance of LSSVM *L*_2 _MKL is always comparable to the best performance. The SIP based LSSVM *L*_2 _MKL classifier has two main "solid advantages": the inherent time complexity is small and the regularization parameter λ can be jointly estimated in the experimental setup. Due to these merits, LSSVM *L*_2 _MKL is a very promising technique for problems pertaining to large scale data fusion.

## Conclusions

This paper compared the effect of optimizing different norms in multiple kernel learning in a systematic framework. The obtained results extend and enrich the statistical framework of genomic data fusion proposed by Lanckriet *et al*. [4,6] and Bach *et al*. [5]. According to the optimization of different norms in the dual problem of SVM, we proposed *L*_∞_, *L*_1_, and *L*_2 _MKL, which are respectively corresponding to the *L*_1 _regularization, average combination, and *L*_2 _regularization of kernel coefficients addressed in the primal problem.

Six real biomedical data sets were investigated in this paper, where *L*_2 _MKL approach was shown advantageous over the *L*_∞ _method. We also proposed a novel and efficient LSSVM *L*_2 _MKL classifier to learn the optimal combination of multiple large scale data sets. All the algorithms implemented in this paper are freely accessible on http://homes.esat.kuleuven.be/~sistawww/bioi/syu/l2lssvm.html.

## Authors' contributions

All authors conceived the project and design. SY performed the theoretical analysis, programmed the algorithms, analyzed the data and wrote the paper. TF investigated SIP and implemented SIP formulations for SVM and LSSVM. AD preprocessed the rectal cancer, endometrial, miscarriage and pregnancy data sets. AD also provided the code of clinical kernel construction. LCT provided the data sources, disease relevant benchmark genes and prostate cancer genes for gene prioritization application. LCT also compared the performance of prioritization on Endeavour system. JS is the promoter of TF. BDM is the promoter of AD and SY. YM is the promoter of SY and LCT. All authors read and approved the manuscript. AD is research assistant of the Fund for Scientific Research - Flanders (FWO-Vlaanderen) JS and YM are professor and BDM a full professor at the Katholieke Universiteit Leuven, Belgium. All authors read and approved the manuscript.

## Appendix

**Algorithm 0.1**: SIP-SVM-MKL(*K*_*j*_, *Y*_*q*_, *C*, *ε*)

Obtain the initial guess 

**while **(Δ*u *>*ε*)

**comment: ***τ *is the indicator of the current loop

**return **

**Algorithm 0.2**: SIP-LSSVM-MKL(*K*_*j*_, *Y*_*q*_, *ε*)

Obtain the initial guess 

**while **(**Δ***u *>*ε*)

**comment**: *τ *is the indicator of the current loop

**return **

## Supplementary Material

Additional file 1 The supplementary material contains (1) Genomic data sources used in experiment 1 and 2; (2) MKL extensions for Weighted SVM and Weighted LSSVM; (3) Kernel functions used in the paper; (4) Optimal kernel coefficients and performance of individual data sources in prostate cancer genes prioritization; (5) Performance of individual kernels in experiment 4; (6) Optimal weights assigned on each individual kernels in Experiment 4; (7) The effect of cost function regularization parameter λ of LSSVM in experiment 4; (8) Experimental results using MKL algorithms based on other norms. Click here for file
