# Supplementary material for: L2-norm multiple kernel learning and its application to biomedical data fusion
Source: BMC Bioinformatics. 2010 Jun 8;11:309. doi: 10.1186/1471-2105-11-309 (PMC2906488; doi:10.1186/1471-2105-11-309)
Supplement: Additional file 1 — The supplementary material contains (1) Genomic data sources used in experiment 1 and 2; (2) MKL extensions for Weighted SVM and Weighted LSSVM; (3) Kernel functions used in the paper; (4) Optimal kernel coefficients and performance of individual data sources in prostate cancer genes prioritization; (5) Performance of individual kernels in experiment 4; (6) Optimal weights assigned on each individual kernels in Experiment 4; (7) The effect of cost function regularization parameter λ of LSSVM in experiment 4; (8) Experimental results using MKL algorithms based on other norms. [file 1471-2105-11-309-S1.PDF]

L_2-norm multiple kernel learning and its application to biomedical data fusion

Shi Yu*[1], Tillmann Falck[2] , Anneleen Daemen[1] , Leon-Charles Tranchevent[1] ,
Johan A.K. Suykens[2] , Bart De Moor[1] and Yves Moreau[1]

[1]Bioinformatics Group, Department of Electrical Engineering, Katholieke Universiteit Leuven, Kasteelpark Arenberg 10, Heverlee B-3001, Belgium

[2]Systems, Models and Control Group, Department of Electrical Engineering, Katholieke Universiteit Leuven, Kasteelpark Arenberg 10, Heverlee B-3001, Belgium

Email: Shi Yu*- shi.yu@esat.kuleuven.be; Tillmann Falck - Tillmann.Falck@esat.kuleuven.be; Anneleen Daemen - Anneleen.Daemen@esat.kuleuven.be; Leon-Charles Tranchevent - Leon-Charles.Tranchevent@esat.kuleuven.be; Johan A.K. Suykens, Johan.Suykens@esat.kuleuven.be; Bart De Moor - Bart.DeMoor@esat.kuleuven.be; Yves Moreau - Yves.Moreau@esat.kuleuven.be;

*Corresponding author

Supplementary material 1 - Genomic data sources used in experiment 1 and 2

Table 1: Genomic data sources used in experiment 1 and 2.

data source	reference	type	features	kernel function
EST	[1]	expressed sequence tagging annotations	167	linear
GO	[2]	GO annotations	8643	linear
Interpro	[3]	annotations	4708	linear
KEGG pathways	[4]	interactions	314	linear
Motif	[5][6]	motif findings	674	linear
Sequence	[7]	amino acid sequences	20	2-mer string
Microarray Son *et al.*	[8]	expression array	158	linear
Microarray Su *et al.*	[9]	expression array	158	linear
Text	[10][11]	gene by term vectors using GO vocabulary	7403	linear

Supplementary material 2 - MKL extensions for Weighted SVM and Weighted LSSVM

Weighted SVM

The conventional SVM does not perform well in the presence of imbalanced data. Weighted SVM was proposed to cope with this problem [12, 13, 14]. As an extension from conventional SVM two different penalty constraints were introduced for the positive and negative classes. The optimization problem

becomes,

$$\boxed{\text{P:}} \qquad \begin{aligned} \underset{\vec{w},b,\xi}{\text{minimize}} \quad & \frac{1}{2}\vec{w}^T\vec{w} + C_+ \sum_{\{i|y_i=+1\}} \xi_i^{k+} + C_- \sum_{\{i|y_i=-1\}} \xi_i^{k-} \\ \text{subject to} \quad & y_i[\vec{w}^T\phi(\vec{x}_i) + b] \geq 1 - \xi_i, \ \ i = 1, ..., N \\ & \xi_i \geq 0, \ \ i = 1, ..., N, \end{aligned} \tag{1}$$

where $\vec{x}_i$ are data samples, $\phi(\cdot)$ is the feature map, y_i are class labels, C_+ and C_- are respectively the penalty coefficients for positive class samples and negative class samples, ξ_i are slack variables, $k+$ and $k-$ are respectively the numbers of slack variables for positive and negative class samples, $\vec{w}$ is the norm vector of the separating hyperplane, and b is the bias. This problem is also convex and can be solved as a dual problem, given by

$$\boxed{\text{D:}} \qquad \begin{aligned} \underset{\vec{\alpha}}{\text{minimize}} \quad & \frac{1}{2}\vec{\alpha}^T Y K Y \vec{\alpha} - \vec{\alpha}^T\vec{1} \\ \text{subject to} \quad & (Y\vec{\alpha})^T\vec{1} = 0 \\ & 0 \leq \alpha_i \leq C_+, \ \ \{\forall i | y_i = +1\} \\ & 0 \leq \alpha_i \leq C_-, \ \ \{\forall i | y_i = -1\}, \end{aligned} \tag{2}$$

where $\vec{\alpha}$ are the dual variables, $Y = diag(y_1, ..., y_N)$, K is the kernel matrix, and C_+ and C_- are two different upperbounds of the box constraints on dual variables correspond to different classes. The value of C_+ and C_- should be predetermined. In practical, one can fix C_- and optimize the performance on training data by varying C_+ [14]. Suppose the dominant class is $+$, then its penalty value C_+ should be smaller than the value of rare class samples. In our paper, the reported results on pregnancy data are obtained by $2C_+ = C_- = 2$.

Weighted SVM MKL

The MKL extension of Weighted SVM is analogous to the MKL extension of the unweighted SVM. The L_∞ MKL for binary class SVM is given by

$$\boxed{\text{D:}} \qquad \begin{aligned} \underset{\gamma,\vec{\alpha}}{\text{minimize}} \quad & \frac{1}{2}\gamma - \vec{\alpha}^T\vec{1} \\ \text{subject to} \quad & (Y\vec{\alpha})^T\vec{1} = 0 \\ & 0 \leq \alpha_i \leq C_+, \ \{\forall i | y_i = +1\} \\ & 0 \leq \alpha_i \leq C_-, \ \{\forall i | y_i = -1\} \\ & \gamma \geq \vec{\alpha}^T Y K_j Y \vec{\alpha}, \ \ \ j = 1, ..., p, \end{aligned} \tag{3}$$

where p is the number of kernels.

The L_2-norm MKL is analogously given by

$$\boxed{\text{D:}} \qquad \begin{aligned} \underset{\eta,\vec{\alpha}}{\text{minimize}} \quad & \frac{1}{2}\eta - \vec{\alpha}^T\vec{1} \\ \text{subject to} \quad & (Y\vec{\alpha})^T\vec{1} = 0 \\ & 0 \leq \alpha_i \leq C_+, \ \{\forall i | y_i = +1\} \\ & 0 \leq \alpha_i \leq C_-, \ \{\forall i | y_i = -1\} \\ & \eta \geq ||\gamma_j||_2, \quad j = 1, ..., p \\ & \gamma_j \geq \vec{\alpha}^T Y K_j Y \vec{\alpha}. \ \ j = 1, ..., p. \end{aligned} \tag{4}$$

Weighted LSSVM

In LSSVM, the cost function can be extended to cope with imbalanced data, given by

$$\begin{aligned} \underset{\vec{w},b,\vec{e}}{\text{minimize}} \quad & \frac{1}{2}\vec{w}^T\vec{w} + \frac{1}{2}\lambda\sum_{i=1}^{N} v_i e_i^2 \\ \text{subject to} \quad & y_i[\vec{w}^T\phi(\vec{x}_i) + b] = 1 - e_i, \ i = 1, ..., N, \end{aligned} \tag{5}$$

where the main difference with unweighted LSSVM is that the least squares terms are weighted for different samples. Suppose $\vec{v} = \{v_1, v_2, ..., v_N\}$ is a vector of weights associated with each sample, taking the conditions for optimality from the Lagrangian, eliminating $\vec{w}, \vec{e}$, defining $\vec{y} = [y_1, ..., y_N]^T$, $Y = diag(y_1, ..., y_N)$ and $W = diag(v_1^{-1}, ..., v_N^{-1})$, the weighted LSSVM can be solved as the following linear system [15]:

$$\left[\begin{array}{c|c} 0 & \vec{y}^T \\ \hline \vec{y} & YKY + W/\lambda \end{array}\right] \left[\begin{array}{c} b \\ \hline \vec{\alpha} \end{array}\right] = \left[\begin{array}{c} 0 \\ \hline \vec{1} \end{array}\right], \tag{6}$$

To improve the robustness of LSSVM when coping with the imbalanced data, a simple way to choose the weighting factors is [16]

$$v_i = \begin{cases} N/2N_+ & \text{if } y_i = +1 \\ N/2N_- & \text{if } y_i = -1 \end{cases} \tag{7}$$

where N_+ and N_- represent the number of positive and negative samples respectively. In our paper, the reported results on pregnancy data are obtained by weighted LSSVM using the setting described above.

Weighted LSSVM MKL

To incorporate multiple kernels in Weighted LSSVM, the QCQP based L_∞ solution is given by (assuming $Y^{-2} = I$)

$$\begin{aligned} \underset{\vec{\alpha},t}{\text{minimize}} \quad & \frac{1}{2}t + \frac{1}{2\lambda}\vec{\beta}^T W \vec{\beta} - \vec{\beta}^T Y^{-1}\vec{1} \\ \text{subject to} \quad & \sum_{i=1}^{N} \beta_i = 0, \\ & t \geq \vec{\beta}^T K_j \vec{\beta}, \; j = 1, ..., p. \end{aligned} \tag{8}$$

where most of the variables are defined the same as in the unweighted version. The weight factor matrix W is defined as same in (6). The L_2-norm approach is analogously formulated as

$$\begin{aligned} \underset{\vec{\alpha},\eta}{\text{minimize}} \quad & \frac{1}{2}\eta + \frac{1}{2\lambda}\vec{\beta}^T W \vec{\beta} - \vec{\beta}^T Y^{-1}\vec{1} \\ \text{subject to} \quad & \sum_{i=1}^{N} \beta_i = 0, \\ & s_j \geq \vec{\beta}^T K_j \vec{\beta}, \; j = 1, ..., p, \\ & \eta \geq ||s_j||_2, \; j = 1, ..., p. \end{aligned} \tag{9}$$

The SIP based formulations for Weighted LSSVM MKL are analogous to the unweighted version, with the only difference that the single kernel weighted LSSVM is solved as the linear system defined in (6).

Supplementary material 3 - Kernel functions used in the paper

Linear Kernel

The linear kernel function is proposed as the inner product of two vector based data samples. Let $K(\vec{x}_i, \vec{x}_j)$ denotes the kernel function for samples $\vec{s}_i$ and $\vec{s}_j$, the linear kernel function is defined as

$$K(\vec{x}_i, \vec{x}_j) = \vec{x}_i^T \vec{x}_j. \tag{10}$$

RBF Kernel

The RBF kernel function is defined as

$$K(\vec{x}_i, \vec{x}_j) = e^{-\frac{||\vec{x}_i - \vec{x}_j||^2}{2\sigma^2}}, \tag{11}$$

where σ is a tuning parameter determining the *width* of the RBF kernel function.

Polynomial Kernel

The polynomial kernel is defined as

$$K(\vec{x}_i, \vec{x}_j) = (\vec{x}_i^T \vec{x}_j + b)^d, \quad b \geq 0 \tag{12}$$

where b is the bias term and, d the degree of the polynomial function. When $b = 0$, the function is also called *homogeneous polynomial kernel*. When $b \neq 0$, it is called *non-homogeneous polynomial kernel*. In our paper, b is set to 1.

Clinical Kernel

The clinical kernel function is proposed for each variable type as in [17]. Let $k(v_i, v_j)$ denotes the kernel function for variable v between patients i and j; $K_v(i,j)$ represents the corresponding individual kernel matrix for variable v; $K(i,j)$ represents the global, heterogeneous kernel matrix.

- *Continuous and ordinal clinical variables:* The same kernel function is proposed for these variable types:

$$k_v(i,j) = \frac{C - |v_i - v_j|}{C}, \tag{13}$$

  where the constant value C is usually defined as the range between maximal value between minimal value of variable v on the training set, given by

$$C = max - min. \tag{14}$$

- *Nominal clinical variables:* For nominal variables, the kernel function between patients i and j is defined as

$$k_v(i,j) = \begin{cases} 1 & \text{if} \quad v_i = v_j \\ 0 & \text{if} \quad v_i \neq v_j \end{cases} \quad . \tag{15}$$

- *Final kernel for clinical data:* Because each individual kernel matrix has been normalized to the interval [0,1], the global, heterogeneous kernel matrix can be defined as the sum of the individual kernel matrices, divided by the total number of clinical variables. This matrix then describes the similarity for a class of patients based on a set of variables of different type.

For example, in the endometrial data set, we would like to calculate the kernel function between two patients i and j for the variables age, number of miscarriages/abortions, and menopausal status, which are respectively continuous, ordinal and nominal variables. Suppose that patient i is 23 years old, has 1 miscarriage and the nominal menopausal status value is 2; patient j is 28 years old, has 2 miscarriage and the menopausal status value is 3. Suppose that, based on the training data, the minimal age is 20 and the maximal age is 100. The minimal miscarriage number is 0 and the maximal number is 5. Then for each variable, the kernel functions between i and j are:

$$k_{age}(i,j) = ((100-20) - |23-28|)/(100-20) = 0.9375 \quad ,$$

$$k_{miscarriage}(i,j) = ((5-0) - |1-2|)/(5-0) = 0.8 \quad ,$$

$$k_{menopausal}(i,j) = 0 \quad .$$

The overall kernel function between patient i and j is given by

$$K(i,j) = \frac{1}{3}(k_{age} + k_{miscarriage} + k_{menopausal}) = 0.5792 \quad .$$

Supplementary material 4 - Optimal kernel coefficients and performance of individual data sources in prostate cancer genes prioritization

Table 2: Results of experiment 2: prioritization of prostate cancer genes by genomic data fusion. For each gene, the best individual data sources are shown in bold. Apparently, the sparse kernel coefficients optimized by L_∞ 1-SVM MKL is too selective thus sometimes the best individual data sources are discarded in integration. In comparison, the L_2 method is good at evenly combining multiple data sources.

Name		EST	GO	Interpro	KEGG	Motif	Sequence	MA1 (Son *et al.*)	MA2 (Su *et al.*)	Text
CPNE	Error AUC	0.5000	0.2929	0.5000	0.4091	0.5000	0.3535	0.0909	0.3636	**0.0505**
	Rank position	58/100	30/100	37/100	50/100	53/100	36/100	10/100	37/100	**6/100**
	L_∞ coefficients	0	0	0	0	0	0	0	0.7561	0.2439
	L_2 coefficients	0.0776	0.3006	0.2726	0.1423	0.2786	0.1789	0.4075	0.5400	0.4917
CDH23	Error AUC	0.5000	0.1212	0.2929	0.5000	0.5000	0.2020	0.2929	**0.0202**	0.0606
	Rank position	71/100	13/100	30/100	89/100	54/100	21/100	30/100	**3/100**	7/100
	L_∞ coefficients	0	0	0	0	0	0	0	0	1
	L_2 coefficients	0.0580	0.3331	0.3143	0.1972	0.2978	0.1823	0.2743	0.4412	0.5968
EHBP1	Error AUC	0.5000	0.5000	0.2424	0.4545	0.4040	0.0707	**0.0505**	0.1414	0.5000
	Rank position	54/100	65/100	11/100	50/100	41/100	8/100	**6/100**	15/100	84/100
	L_∞ coefficients	0	0	0	0	0	0	0	0.1905	0.8095
	L_2 coefficients	0.0733	0.3638	0.3013	0.1832	0.2921	0.1653	0.3833	0.4619	0.5148
MSMB	Error AUC	0.1616	0.3737	0.5000	0.5000	0.0606	0.3030	**0.0202**	0.3333	0.0303
	Rank position	15/100	38/100	60/100	92/100	7/100	31/100	**3/100**	34/100	4/100
	L_∞ coefficients	0	0	0	0	0	0	1	0	0
	L_2 coefficients	0.0949	0.2936	0.2014	0.1198	0.2242	0.1256	0.7389	0.3198	0.3683
KLK3	Error AUC	0.1616	0.5000	**0.2475**	0.4545	0.5000	0.5000	0.3535	0.3535	0.5000
	Rank position	17/100	63/100	**19/100**	20/100	87/100	77/100	36/100	36/100	94/100
	L_∞ coefficients	0	0	0	0	0	0	0.9373	0	0.0627
	L_2 coefficients	0.1666	0.2534	0.3097	0.1193	0.2247	0.2345	0.5921	0.4149	0.4120
JAZF1	Error AUC	0.4242	0.1212	0.5000	0.4444	**0.0606**	0.3131	0.2828	0.0707	0.1010
	Rank position	43/100	13/100	86/100	37/100	**7/100**	32/100	29/100	8/100	11/100
	L_∞ coefficients	0	0	0	0	0	0	0.3541	0	0.6459
	L_2 coefficients	0.1301	0.2813	0.2990	0.1645	0.3004	0.1924	0.4661	0.4300	0.5081
LMTK2	Error AUC	0.5000	0.5000	0.1212	0.4293	0.5000	**0**	0.5000	0.5000	0.3232
	Rank position	79/100	58/100	13/100	29/100	70/100	**1/100**	71/100	65/100	24/100
	L_∞ coefficients	0	0	0	0	0	0	0	0	1
	L_2 coefficients	0.1134	0.3117	0.2836	0.1875	0.2777	0.2029	0.3584	0.4305	0.5850
IL16	Error AUC	**0**	0.3939	0.5000	0.5000	0.5000	0.3636	0.0202	0.1212	0.0202
	Rank position	**1/100**	40/100	73/100	91/100	51/100	37/100	3/100	13/100	3/100
	L_∞ coefficients	0	0	0	0	0	0	0.4991	0.2808	0.2201
	L_2 coefficients	0.0937	0.3723	0.2379	0.1456	0.2035	0.1502	0.5162	0.4918	0.4500
CTBP2	Error AUC	0.5000	**0.0808**	0.5000	0.4546	0.5000	0.1313	0.5000	0.5000	0.5000
	Rank position	69/100	**9/100**	45/100	49/100	51/100	14/100	61/100	48/100	72/100
	L_∞ coefficients	0	0	0	0	0	0	0	0.7801	0.2199
	L_2 coefficients	0.0726	0.3348	0.2368	0.1391	0.2419	0.1375	0.4189	0.5802	0.4665

Supplementary material 5 - Performance of individual kernels in Experiment 4

Table 3: Performance of individual kernels in Experiment 4. For each combination of data set and algorithm, the best individual kernels are shown in bold. For each data set across different single kernel algorithms, the best results are underlined. The best MKL performance is also shown for comparison. Obviously, MKL performance is comparable to the results of best individual kernels.

Data Set	Classifier	Kernel	Error of AUC (mean)	Error of AUC (std.)
endometrial disease	LSSVM	linear	0.2820	0.0175
		RBF1	0.2923	0.0131
		RBF2	0.2844	0.0118
		RBF3	0.2915	0.0119
		POLY1	0.3223	0.0088
		POLY2	0.3226	0.0109
		POLY3	0.3183	0.0128
		Clinical	**0.2126**	**0.0098**
	SVM	linear	0.2816	0.0192
		RBF1	0.2971	0.0112
		RBF2	0.2817	0.0098
		RBF3	0.2877	0.0133
		POLY1	0.3271	0.0141
		POLY2	0.3214	0.0130
		POLY3	0.3225	0.0135
		Clinical	<u>**0.2021**</u>	<u>**0.0084**</u>
	best MKL classifier		0.2353	0.0133
miscarriage	LSSVM	linear	0.2410	0.0022
		RBF1	<u>**0.1993**</u>	<u>**0.0042**</u>
		RBF2	0.2114	0.0029
		RBF3	0.2182	0.0030
		POLY1	0.2637	0.0020
		POLY2	0.2607	0.0023
		POLY3	0.2593	0.0019
		Clinical	0.2301	0.0026
	SVM	linear	0.2781	0.0065
		RBF1	**0.2098**	**0.0029**
		RBF2	0.2272	0.0042
		RBF3	0.2352	0.0037
		POLY1	0.2771	0.0013
		POLY2	0.2741	0.0023
		POLY3	0.2713	0.0016
		Clinical	0.2441	0.0035
	best MKL classifier		0.1975	0.0037
pregnancy	Weighted LSSVM	linear	0.1666	0.0118
		RBF1	0.1763	0.0142
		RBF2	0.1990	0.0146
		RBF3	0.2137	0.0170
		POLY1	0.2836	0.0154
		POLY2	0.2639	0.0169
		POLY3	0.2382	0.0180
		Clinical	<u>**0.1160**</u>	<u>**0.0092**</u>
	Weighted SVM	linear	**0.1461**	**0.0074**
		RBF1	0.2127	0.0187
		RBF2	0.2017	0.0254
		RBF3	0.1906	0.0221
		POLY1	0.1478	0.0184
		POLY2	0.1541	0.0204
		POLY3	0.1594	0.0179
		Clinical	0.1601	0.0188
	best MKL classifier		0.1165	0.0100

Supplementary material 6 - Optimal weights assigned on each individual kernels in Experiment 4

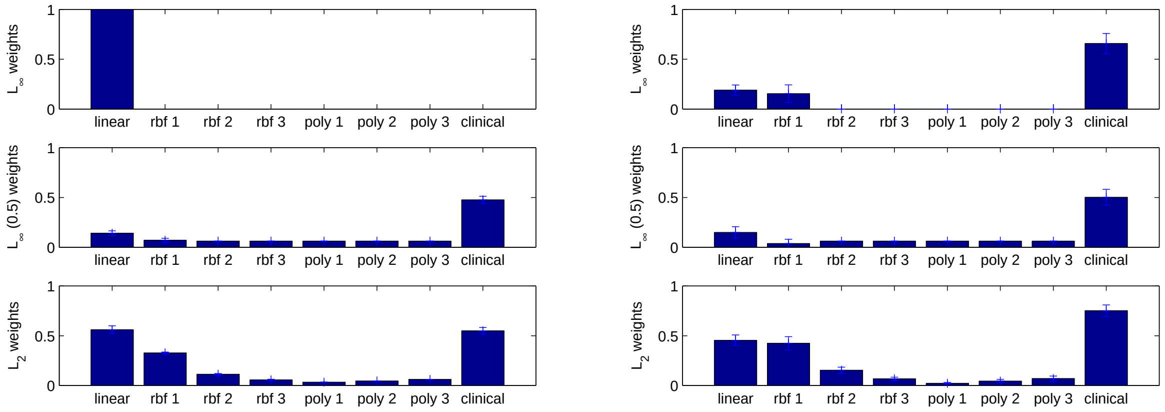

Figure 1: The kernel coefficients optimized on endometrial disease data set. Figure on the left shows coefficients of LSSVM MKL. Figure on the right are coefficients of SVM MKL.

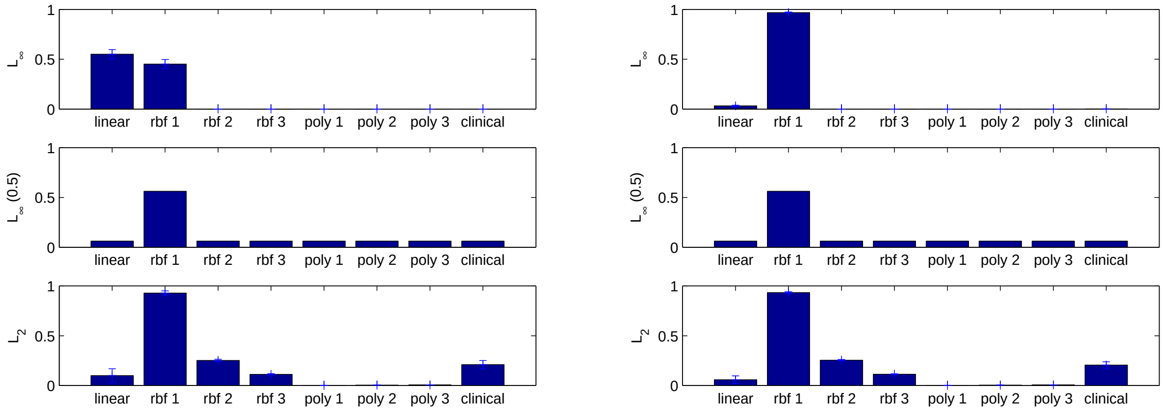

Figure 2: The kernel coefficients optimized on miscarriage data set. Figure on the left shows coefficients of LSSVM MKL. Figure on the right are coefficients of SVM MKL.

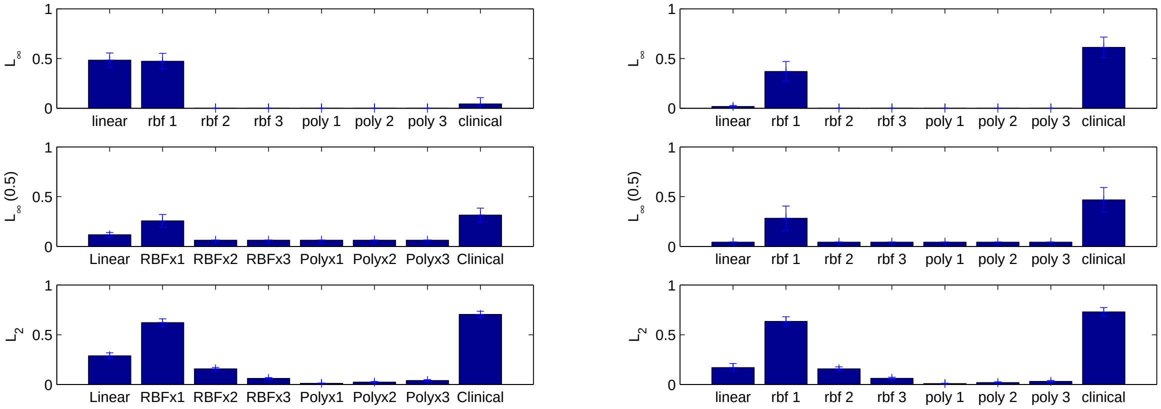

Figure 3: The kernel coefficients optimized on pregnancy data set. Figure on the left shows coefficients of weighted LSSVM MKL. Figure on the right are coefficients of weighted SVM MKL.

Supplementary material 7 - The effect of cost function regularization parameter λ of LSSVM in Experiment 4

To show the effect of λ value in LSSVM MKL and weighted LSSVM MKL, we tried 21 fixed λ values from $\{2^{-10}, 2^{-9}, ..., 2^{9}, 2^{10}\}$. For each fixed λ value, we divide the data set into 3-fold and use two folds to train the classifier and evaluate the performance on the other fold. In each repetition, we average the generalization performance on the 3 folds. On each data set, we repeat 3-fold evaluation 20 times and the mean values and standard deviations are plotted in the figure. As shown in Figure 4, 5, and 6, the λ value significantly affects the performance of LSSVM MKL classifier. The optimal λ value obviously depends on data set so it is hard to predefine its value by “rule of thumb”.

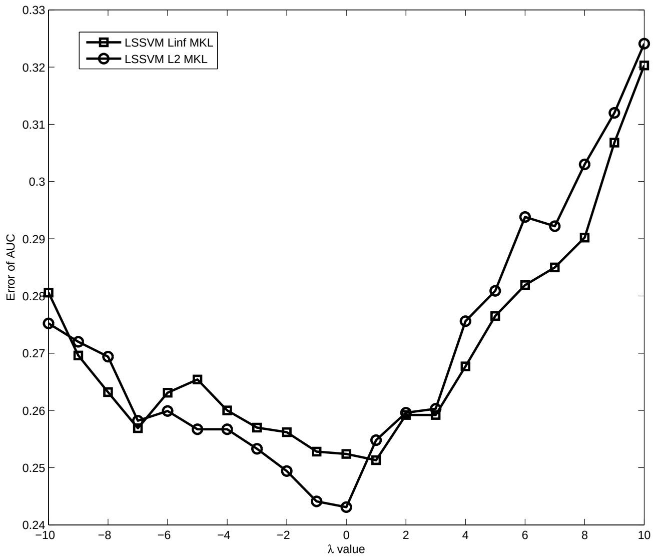

Figure 4: The performance of LSSVM MKL classifiers varied by various λ values on endometrial disease data set

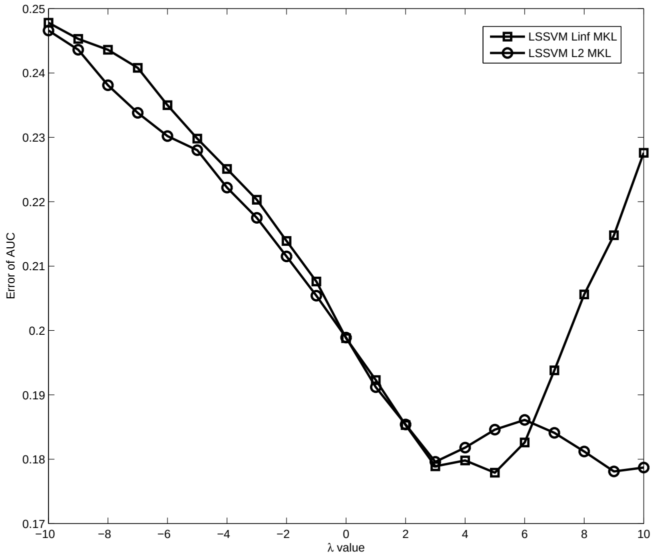

Figure 5: The performance of LSSVM MKL classifiers varied by various λ values on miscarriage data set

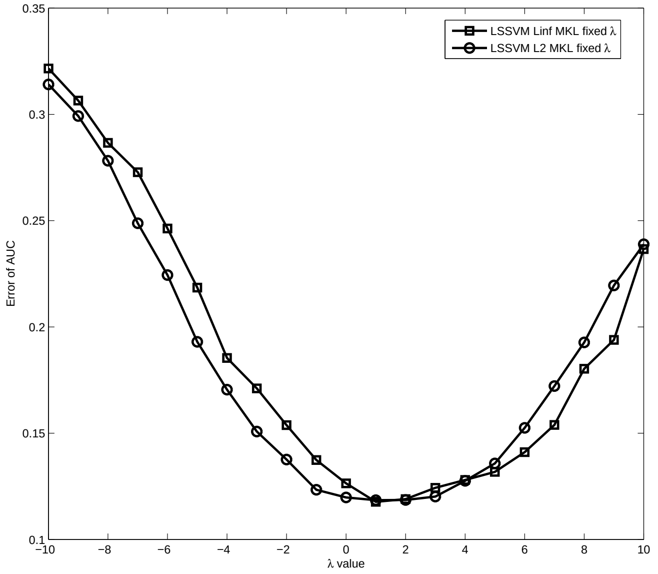

Figure 6: The performance of Weighted LSSVM MKL classifiers varied by various λ values on pregnancy data set

Supplementary material 8 - Experimental results using MKL algorithms based on other norms

We extended all the algorithms to other norms by adding a new free parameter (n for SOCP based formulations, m for SIP based formulations). For each algorithm, we tried 5 new norm values (n=1.5, 1.3333,1.25,1.2, and 1.16667; n=3,4,5,6,7) on all the 4 biomedical experimental data sets. The performance is shown from Table 4 to Table 7.

Table 4: Results of experiment 1 with other norms: prioritization of 620 disease relevant genes by genomic data fusion

	Error of AUC (mean)	Error of AUC (std.)
L_∞	0.0923	0.0035
$L_\infty(0.5)$	0.0806	0.0033
L_1	0.0908	0.0042
L_2	**0.0780**	0.0034
$L_{1.5}$	0.0865	0.0046
$L_{1.3333}$	0.0889	0.0047
$L_{1.25}$	0.0903	0.0047
$L_{1.2}$	0.0912	0.0048
$L_{1.1667}$	0.0919	0.0048

The error of AUC values is evaluated by LOO validation in 20 random repetitions. The best performance (L_2) is shown in bold. As shown, the performance of other norms is not comparable to the L_2 one.

Figure 7: The optimal kernel coefficients of experiment 1 with other norms: prioritization of 620 disease relevant genes by genomic data fusion

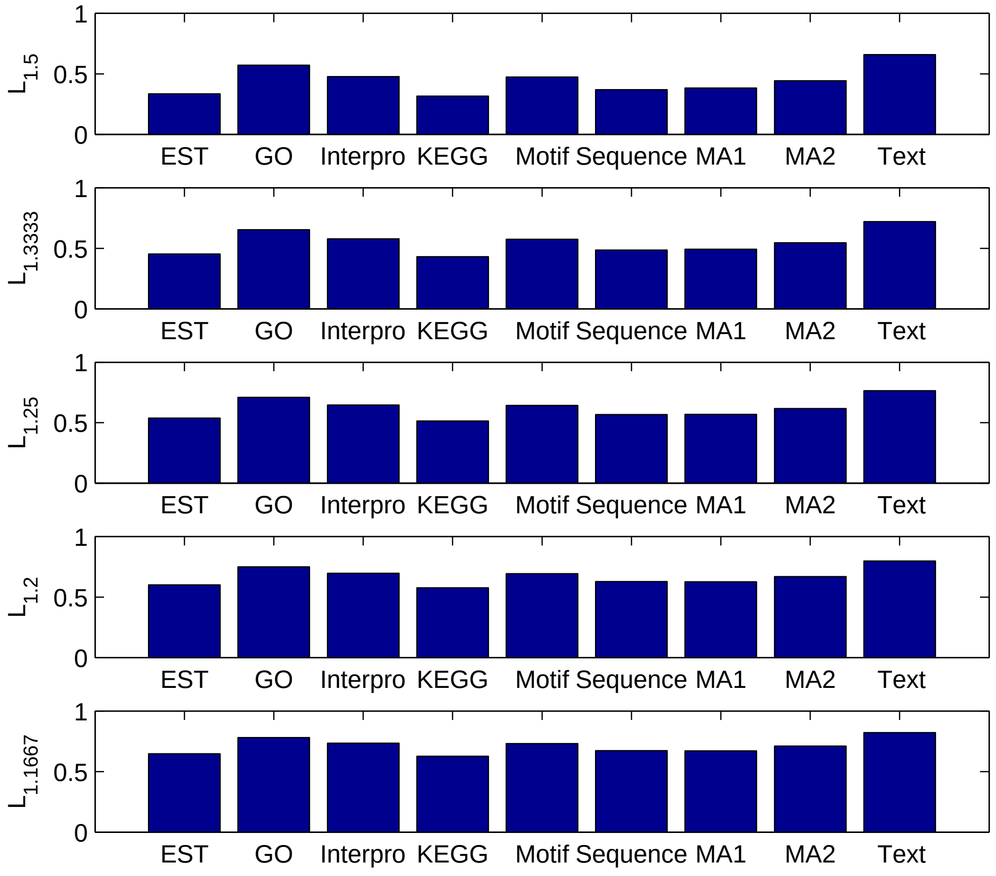

The L_∞, $L_\infty(0.5)$,L_1, and L_2 coefficients are already shown in the main manuscript. The coefficients of other norms are presented here. As shown, when the norm of the dual problem is closer to 1 (the norm of the regularization term in the primal problem is higher), the coefficients are more evenly distributed on multiple kernels.

Table 5: Results of experiment 2 with other norms: prioritization of prostate cancer genes by genomic data fusion

Name	L_∞	$L_\infty(0.5)$	L_1	L_2	$L_{1.5}$	$L_{1.3333}$	$L_{1.25}$	$L_{1.2}$	$L_{1.1667}$	Endeavour
CPNE	0.3030<br>31/100	0.2323<br>24/100	**0.1010**<br>**11/100**	*0.1212*<br>*13/100*	0.1111<br>12/10	0.1111<br>12/10	0.1111<br>12/10	0.1111<br>12/10	0.1212<br>13/10	-<br>70/100
CDH23	0.0606<br>7/100	0.0303<br>4/100	*0.0202*<br>*3/100*	**0.0101**<br>**2/100**	0.0202<br>3/10	0.0202<br>3/10	0.0202<br>3/10	0.0202<br>3/10	0.0202<br>3/10	-<br>78/100
EHBP1	0.5354<br>54/100	0.5152<br>52/100	**0.3434**<br>**35/100**	*0.3939*<br>*40/100*	0.3737<br>38/100	0.3636<br>37/100	0.3535<br>36/100	0.3535<br>36/100	0.3535<br>36/100	-<br>57/100
MSMB	**0.0202**<br>**3/100**	**0.0202**<br>**3/100**	0.0505<br>6/100	*0.0303*<br>*4/100*	0.0404<br>5/100	0.0505<br>6/100	0.0505<br>6/100	0.0505<br>6/100	0.0505<br>6/100	-<br>69/100
KLK3	0.3434<br>35/100	0.3535<br>36/100	*0.2929*<br>*30/100*	*0.2929*<br>*30/100*	0.3030<br>31/100	0.3030<br>31/100	0.3030<br>31/100	0.3030<br>31/100	0.3030<br>31/100	-<br>**28/100**
JAZF1	*0.0505*<br>*6/100*	**0.0202**<br>**3/100**	**0.0202**<br>**3/100**	**0.0202**<br>**3/100**	**0.0202**<br>**3/100**	**0.0202**<br>**3/100**	**0.0202**<br>**3/100**	**0.0202**<br>**3/100**	**0.0202**<br>**3/100**	-<br>7/100
LMTK2	*0.3131*<br>*32/100*	0.4646<br>47/100	0.8081<br>81/100	0.7677<br>77/100	0.7879<br>78/100	0.8081<br>79/100	0.8081<br>81/100	0.8081<br>81/100	0.8081<br>81/100	-<br>81/100
IL16	**0**<br>**1/100**	*0.0101*<br>*2/100*	0.0303<br>4/100	*0.0101*<br>*2/100*	0.0202<br>3/100	0.0303<br>4/100	0.0303<br>4/100	0.0303<br>4/100	0.0303<br>4/100	-<br>72/100
CTBP2	0.8283<br>83/100	0.5758<br>58/100	*0.6364*<br>*64/100*	0.6869<br>69/100	0.6667<br>67/100	0.6566<br>66/100	0.6465<br>65/100	0.6465<br>65/100	0.6465<br>65/100	-<br>**38/100**

To compare the performance, we show all the old results together with results obtained on other norms. Obviously, the new norms do not bring any significant advantage in prostate cancer gene ranking. Only on JAZF1 gene, the other norms have same ranking results with L_1 and L_2. For all the other genes, the performance of other norms is not comparable to L_1 or L_2.

Table 6: Results of experiment 3 with other norms: classification of patients in rectal cancer clinical decision using microarray and proteomics data sets

	LSSVM $L_{1.5}$					SVM $L_{1.5}$				
	14p	15p	16p	17p	18p	14p	15p	16p	17p	18p
24g	0.0552	**0.0487**	*0.0649*	*0.0779*	*0.0714*	0.0877	0.0747	0.0909	0.0909	0.0942
25g	0.0422	**0.0325**	0.0487	0.0519	0.0552	0.0682	0.0617	0.0779	0.0747	0.0779
26g	0.0422	**0.0357**	0.0519	0.0617	0.0617	0.0682	0.0552	0.0714	0.0682	0.0682
27g	0.0390	0.0455	0.0552	0.0552	*0.0617*	0.0714	0.0617	0.0747	0.0682	0.0682
28g	**0.0390**	**0.0292**	0.0455	0.0552	*0.0519*	0.0682	0.0649	0.0714	0.0682	0.0682

	LSSVM $L_{1.3333}$					SVM $L_{1.3333}$				
	14p	15p	16p	17p	18p	14p	15p	16p	17p	18p
24g	0.0519	**0.0487**	*0.0649*	*0.0779*	*0.0714*	0.0812	0.0747	0.0812	0.0844	0.0844
25g	0.0422	**0.0325**	*0.0422*	0.0519	0.0552	0.0649	0.0617	0.0747	0.0714	0.0714
26g	*0.0390*	**0.0357**	0.0487	0.0584	*0.0584*	0.0649	0.0552	0.0714	0.0682	0.0682
27g	0.0422	0.0422	0.0552	0.0552	*0.0617*	0.0682	0.0584	0.0714	0.0682	0.0682
28g	**0.0390**	**0.0292**	**0.0422**	0.0552	**0.0487**	0.0617	0.0584	0.0682	0.0682	0.0682

	LSSVM $L_{1.25}$					SVM $L_{1.25}$				
	14p	15p	16p	17p	18p	14p	15p	16p	17p	18p
24g	0.0519	**0.0487**	*0.0649*	*0.0779*	**0.0682**	0.0779	0.0649	0.0747	*0.0779*	0.0812
25g	**0.0357**	**0.0325**	**0.0390**	*0.0487*	0.0552	0.0649	0.0552	0.0682	0.0714	0.0714
26g	**0.0357**	**0.0357**	*0.0455*	**0.0455**	**0.0455**	0.0584	0.0519	0.0682	0.0682	0.0682
27g	**0.0357**	*0.0390*	0.0519	0.0552	*0.0617*	0.0682	0.0584	0.0714	0.0682	0.0682
28g	**0.0390**	**0.0292**	**0.0422**	*0.0519*	**0.0487**	0.0617	0.0584	0.0682	0.0682	0.0682

	LSSVM $L_{1.2}$					SVM $L_{1.2}$				
	14p	15p	16p	17p	18p	14p	15p	16p	17p	18p
24g	0.0519	**0.0487**	**0.0617**	*0.0779*	**0.0682**	0.0779	0.0649	0.0747	*0.0779*	0.0812
25g	**0.0357**	**0.0325**	**0.0390**	*0.0487*	0.0552	0.0649	0.0552	0.0682	0.0714	0.0714
26g	**0.0357**	**0.0357**	*0.0455*	0.0552	*0.0584*	0.0649	0.0519	0.0714	0.0682	0.0682
27g	**0.0357**	*0.0390*	*0.0487*	0.0552	*0.0617*	0.0682	0.0584	0.0714	0.0682	0.0682
28g	**0.0390**	**0.0292**	**0.0422**	*0.0519*	**0.0487**	0.0617	0.0584	0.0682	0.0682	0.0682

	LSSVM $L_{1.1667}$					SVM $L_{1.1667}$				
	14p	15p	16p	17p	18p	14p	15p	16p	17p	18p
24g	0.0519	**0.0487**	**0.0617**	*0.0779*	**0.0682**	0.0779	0.0617	0.0747	*0.0779*	0.0812
25g	**0.0357**	**0.0325**	**0.0390**	*0.0487*	*0.0519*	0.0649	0.0519	0.0682	0.0714	0.0714
26g	**0.0357**	**0.0357**	**0.0422**	0.0519	*0.0584*	0.0649	0.0519	0.0714	0.0682	0.0682
27g	**0.0357**	*0.0390*	**0.0455**	*0.0519*	*0.0617*	0.0682	0.0584	0.0714	0.0682	0.0682
28g	**0.0390**	**0.0292**	**0.0422**	*0.0519*	**0.0487**	0.0617	0.0584	0.0682	0.0682	0.0682

The MKL classifiers based other norms perform well on the data in experimental 3, yielding the best performance of all approaches (Error of AUC 0.0292). The performance is compared with L_∞, $L_\infty(0.5)$, L_1, and L_2 MKL using same number of selected genes and proteins (Table 6 in the main manuscript). For the same number of genes and proteins, the best performance of all classifiers is shown in **bold**. The second best one is shown in *italic*. Due to the large number of combinations of genes and proteins, we only show the best 25 combinations in the table. The performance obtained in these 25 combinations is shown as **underlined**. As shown, when using other norms, LSSVM MKL still performs better than SVM MKL.

The performance obtained with these new norms is generally better than L_∞, L_1, and L_2 norms.

Table 7: Results of experiment 4 data set I with other norms: classification of endometrial disease patients using multiple kernels derived from clinical data

Classifier	Mean - error of AUC	Std. - error of AUC
**LSSVM L_∞ (0.5) MKL**	**0.2353**	**0.0133**
**SVM L_∞ (0.5) MKL**	**0.2388**	**0.0178**
**SVM L_∞ MKL**	**0.2417**	**0.0165**
LSSVM L_2 MKL	0.2456	0.0124
SVM L_2 MKL	0.2489	0.0178
SVM L_1 MKL	0.2513	0.0144
LSSVM L_1 MKL	0.2574	0.0189
LSSVM L_∞ MKL	0.2678	0.0130
LSSVM $L_{1.5}$ MKL	0.2427	0.0107
LSSVM $L_{1.3333}$ MKL	0.2446	0.0100
LSSVM $L_{1.25}$ MKL	0.2466	0.0114
LSSVM $L_{1.2}$ MKL	0.2475	0.0115
LSSVM $L_{1.1667}$ MKL	0.2477	0.0142
SVM $L_{1.5}$ MKL	0.2360	0.0082
SVM $L_{1.3333}$ MKL	0.2375	0.0081
SVM $L_{1.25}$ MKL	0.2373	0.0085
SVM $L_{1.2}$ MKL	0.2368	0.0086
SVM $L_{1.1667}$ MKL	0.2369	0.0089

The λ parameter of LSSVM MKL classifiers are estimated jointly in MKL. The C parameter of SVM MKL is set to 1.

Table 8: Results of experiment 4 data set II with other norms: classification of miscarriage patients using multiple kernels derived from clinical data

Classifier	Mean - error of AUC	Std. - error of AUC
SVM L_2 MKL	0.1975	0.0037
LSSVM L_2 MKL	0.2002	0.0049
LSSVM L_∞ (0.5) MKL	0.2027	0.0045
SVM L_∞ MKL	0.2109	0.0040
SVM L_∞ (0.5) MKL	0.2168	0.0040
LSSVM L_1 MKL	0.2132	0.0029
SVM L_1 MKL	0.2297	0.0038
LSSVM L_∞ MKL	0.2319	0.0015
LSSVM $L_{1.5}$ MKL	0.1892	0.0081
LSSVM $L_{1.3333}$ MKL	0.1921	0.0096
LSSVM $L_{1.25}$ MKL	0.1906	0.0074
LSSVM $L_{1.2}$ MKL	0.1927	0.0080
LSSVM $L_{1.1667}$ MKL	0.1882	0.0064
SVM $L_{1.5}$ MKL	0.2116	0.0050
SVM $L_{1.3333}$ MKL	0.2102	0.0042
SVM $L_{1.25}$ MKL	0.2091	0.0056
SVM $L_{1.2}$ MKL	0.2077	0.0038
SVM $L_{1.1667}$ MKL	0.2093	0.0040

The λ parameter of LSSVM MKL classifiers are estimated jointly in MKL. The C parameter of SVM MKL is set to 1.

Table 9: Results of experiment 4 data set III with other norms: classification of PUL patients using multiple kernels derived from clinical data

Classifier	Mean - error of AUC	Std. - error of AUC
Weighted LSSVM L_2 MKL	0.1165	0.0100
Weighted LSSVM L_1 MKL	0.1243	0.0171
Weighted LSSVM L_∞ (0.5) MKL	0.1290	0.0206
Weighted SVM L_2 MKL	0.1499	0.0248
Weighted SVM L_∞ MKL	0.1552	0.0210
Weighted SVM L_∞ (0.5)	0.1551	0.0153
Weighted SVM L_1 MKL	0.1594	0.0162
Weighted LSSVM L_∞ MKL	0.1651	0.0174
Weighted LSSVM $L_{1.5}$ MKL	0.1086	0.0067
Weighted LSSVM $L_{1.3333}$ MKL	0.1076	0.0069
Weighted LSSVM $L_{1.25}$ MKL	0.1068	0.0070
Weighted LSSVM $L_{1.2}$ MKL	0.1112	0.0129
Weighted LSSVM $L_{1.1667}$ MKL	0.1099	0.0100
Weighted SVM $L_{1.5}$ MKL	0.1244	0.0152
Weighted SVM $L_{1.3333}$ MKL	0.1213	0.0107
Weighted SVM $L_{1.25}$ MKL	0.1234	0.0109
Weighted SVM $L_{1.2}$ MKL	0.1228	0.0141
Weighted SVM $L_{1.1667}$ MKL	0.1199	0.0137

The λ parameter of LSSVM MKL classifiers are estimated jointly in MKL. The C parameter of SVM MKL is set to 1.

References

1. Flicek P, Aken BL, Beal K, Ballester B, Caccamo M, Chen Y, Clarke L, Caotes G, Gunningham F, Cutts T, Down T, Dyer SC, Eyre T, Fitzgerald S, Fernandez-Banet J, Gräf S, Haider S, Hammond R, Holland R, Howe KL, Howe K, Johnson N, Jenkinson A, Kähäri A, Keefe D, Kokocinski F, Kulesha E, Lawson D, Longden I, Megy K, Meidl P, Overduin B, Parker A, Pritchard B, Prlic A, Rice S, Rios D, Schuster M, Sealy I, Slater G, Smedley D, Spudich G, Trevanion S, Vilella AJ, Vogel J, White S, Wood M, Birney E, T C, Curwen V, Durbin R, Fernandez-Suarez XM, Herrero J, Hubbard TJP, Kasprzyk A, Proctor G, Smith J, Ureta-Vidal A, Searle S: **Ensembl 2008**. *Nucleic Acids Research* 2007, **36**:D707–714.
2. Ashburner M, Ball CA, Blake JA, Botstein D, Butler H, Cherry JM, Davis AP, Dolinski K, Dwight SS, Eppig JT, Harris MA, Hill DP, Issel-Tarver L, Kasarskis A, Lewis S, Matese JC, Richardson JE, Ringwald M, Rubin GM, Sherlock G: **Gene ontology: tool for the unification of biology. The Gene Ontology Consortium**. *Nature Genetics* 2000, **25**:25–29.
3. Mulder NJ, Apweiler R, Attwood TK, Bairoch A, Bateman A, Binns D, Bork P, Buillard V, Cerutti L, Copley R, Courcelle E, Das U, Daugherty L, Dibley M, Finn R, Fleischmann W, Gough J, Haft D, Hulo N, Hunter S, Kahn D, Kanapin A, Kejariwal A, Labarga A, Langendijk-Genevaux PS, Lonsdale D, Lopez R, Letunic I, Madera M, Maslen J, McAnulla C, McDowall J, Mistry J, Mitchell A, Nikolskaya AN, Orchard S, Orengo C, Petryszak R, Selengut JD, Sigrist CJA, Thomas PD, Valentin F, Wilson D, Wu CH, Yeats C: **New developments in the InterPro database**. *Nucleic Acids Research* 2007, **35**:D224–D228.
4. Kanehisa M, Araki M, Goto S, Hattori M, Hirakawa M, Itoh M, Katayama T, Kawashima S, Okuda S, Tokimatsu T, Yamanishi Y: **KEGG for linking genomes to life and the environment**. *Nucleic Acids Research* 2008, **36**:D480–D484.
5. Aerts S, Van Loo P, Thijs G, Mayer H, Martin Rd, Moreau Y, De Moor B: **TOUCAN 2: the all-inclusive open source workbench for regulatory sequence analysis**. *Nucleic Acids Research* 2005, **33**:W393–W396.

6. Matys V, Fricke E, Geffers R, Göß ling E, Haubrock M, Hehl R, Hornischer K, Karas D, Kel AE, Kel-Margoulis OV, Kloos DU, Land S, Lewicki-Potapov B, Michael H, Münch R, Reuter I, Rotert S, Saxel H, Scheer M, Thiele S, Wingender E: **TRANSFAC: transcriptional regulation, from patterns to profiles**. *Nucleic Acids Research* 2003, **31**:374–378.
7. Ye J, McGinnis S, Madden TL: **BLAST: improvements for better sequence analysis**. *Nucleic Acids Research* 2006, **34**:W6–W9.
8. Son CG, Bilke S, Davis S, Greer BT, Wei JS, Whiteford CC, Chen QR, Cenacchi N, Khan J: **Database of mRNA gene expression profiles of multiple human organs**. *Genome Research* 2005, **15**(3):443–450.
9. Su AI, Cooke MP, Ching KA, Hakak Y, Walker J, Wiltshire T, Orth AP, Vega RG, Sapinoso LM, Moqrich A, Patapoutian A, Hampton GM, Schultz PG, B HJ: **Large-scale analysis of the human and mouse transcriptomes**. *PNAS* 2002, **99**(7):4465–4470.
10. Yu S, Van Vooren S, Tranchevent L, De Moor B, Moreau Y: **Comparison of vocabularies, representations and ranking algorithms for gene prioritization by text mining**. *Bioinformatics* 2008, **24**(16):i119–i125.
11. Yu S, Tranchevent L, De Moor B, Moreau Y: **Gene prioritization and clustering by multi-view text mining**. *BMC Bioinformatics* 2010, **11**(28):1–48.
12. Osuna E, Freund R, Girosi F: **Support vector machines: Training and applications**. *Tech. Rep. AIM-1602* 1997.
13. Veropoulos K, N C, C C: **Controlling the sensitivity of support vector machines**. *in Proc. of the IJCAI 99* 199, :55–60.
14. Zheng Y, Yang X, Beddoe G: **Reduction of False Positives in Polyp Detection Using Weighted Support Vector Machines**. *in Proc. of the 29th Annual International Conference of the IEEE Engineering in Medicine and Biology Society (EMBC)* 2007, :4433–4436.
15. Suykens JAK, De Brabanter J, Lukas L, Vandewalle J: **Weighted least squares support vector machines : robustness and sparse approximation**. *Neurocomputing, Special issue on fundamental and information processing aspects of neurocomputing* 2002, **48**(1-4):85–105.
16. Cawley GC: **Leave-One-Out Cross-Validation Based Model Selection Criteria for Weighted LS-SVMs**. *in Proc. of 2006 International Joint Conference on Neural Networks* 2006, :1661–1668.
17. Daemen A, De Moor B: **Development of a kernel function for clinical data**. *in Proc. of the 31th Annual International Conference of the IEEE Engineering in Medicine and Biology Society (EMBC)* 2009, :5913–5917.
